# Essential Oil from the Leaves of *Annona neoinsignis* H. Rainer (Annonaceae) Against Liver Cancer: In Vitro and In Vivo Studies

**DOI:** 10.3390/molecules30142971

**Published:** 2025-07-15

**Authors:** Melissa P. Souza, Maria V. L. de Castro, Gabriela A. da C. Barbosa, Sabrine G. Carvalho, Amanda M. R. M. Coelho, Rosane B. Dias, Milena B. P. Soares, Emmanoel V. Costa, Daniel P. Bezerra

**Affiliations:** 1 Postgraduate Program in Chemistry, Institute of Exact Sciences, Federal University of Amazonas (UFAM), Manaus 69080-900, Amazonas, Brazil; melissa.souza@ufam.edu.br; 2 Department of Chemistry, Institute of Exact Sciences, Federal University of Amazonas (UFAM), Manaus 69080-900, Amazonas, Brazil; 3 Gonçalo Moniz Institute, Oswaldo Cruz Foundation (IGM-FIOCRUZ/BA), Salvador 40296-710, Bahia, Brazil; mvictorialimac@gmail.com (M.V.L.d.C.); gabibarbosa.alves@gmail.com (G.A.d.C.B.); sabrinegama17@gmail.com (S.G.C.); amandaribeirocoelho@hotmail.com (A.M.R.M.C.); rosanebd@gmail.com (R.B.D.); milena.soares@fiocruz.br (M.B.P.S.); 4 Department of Biological Sciences, State University of Feira de Santana, Feira de Santana 44036-900, Bahia, Brazil; 5 SENAI Institute for Innovation in Advanced Health Systems, SENAI CIMATEC, Salvador 41650-010, Bahia, Brazil

**Keywords:** *Annona neoinsignis*, essential oil, liver cancer, antitumor

## Abstract

*Annona neoinsignis* H. Rainer (Annonaceae) is a tree native to the Amazon rainforest. Its fruits are also suitable for human consumption in their natural state or are processed to make desserts. In this work, we characterized the chemical composition of the essential oil (EO) from the leaves of *A. neoinsignis* and evaluated its anti-liver-cancer potential via in vitro and in vivo approaches. Chemical composition analysis revealed β-elemene, (*E*)-caryophyllene, germacrene D, and germacrene B as the main constituents. The EO had IC_50_ values ranging from 12.28 to 37.50 μg/mL for B16-F10 cells and MCF-7 cells, whereas an IC_50_ value of >50 μg/mL was found for noncancerous MRC-5 cells. DNA fragmentation, YO-PRO-1 staining, and loss of mitochondrial transmembrane potential were detected in EO-treated HepG2 cells, indicating the induction of apoptosis. Significant in vivo growth inhibition of 53.7% was observed in mice bearing HepG2 cell xenografts treated with EO at a dosage of 40 mg/kg. These data suggest that EO from *A. neoinsignis* leaves is a drug source for liver cancer.

## 1. Introduction

Liver cancer is the third deadliest cancer and the sixth most common cancer worldwide, with an estimated 757,948 deaths and 865,269 new cases in 2022 [1]. By 2040, there will be an estimated 1,392,474 new cases of liver cancer each year [2].

In recent years, several new therapies have been approved to treat advanced liver cancer, including immune checkpoint inhibitors (e.g., atezolizumab, durvalumab, nivolumab, and pembrolizumab), multiple kinase inhibitors (e.g., sorafenib, regorafenib, lenvatinib, and cabozantinib), and angiogenesis inhibitors (bevacizumab and ramucirumab) [3,4,5,6]. On the other hand, although new therapeutic options have been recently approved, current treatments for patients with liver cancer have limited success, so new drugs are urgently needed.

*Annona neoinsignis* H. Rainer (synonyms *Rollinia insignis* R. E. Fr. and *Rollinia insignis var. pallida* R. E. Fr.) (Annonaceae), popularly known in Peru as ‘loreto’ or in Brazil as ‘envira-bobó’, ‘envireira-bobó’, ‘araticum do mato’, ‘cortição’, and ‘cortiça crespa’, is a tree native to the Amazon rainforest. This tree is suitable for both urban landscaping and forest restoration, as its fruits serve as food for birds and other terrestrial animals. Its fruits are also suitable for human consumption in their natural state or are processed to make mousses, ice creams, doughs, or fillings for cakes and cookies [7,8,9,10,11]. Despite this, only one scientific study was conducted with this plant, in which naphthalene was detected as the main volatile constituent of its flowers [12].

Essential oils (EOs) from different *Annona* species, including *Annona senegalensis* Pers., *Annona pickelii* (Diels) H. Rainer, *Annona salzmannii* A. DC., *Annona vepretorum* Mart., *Annona glabra* L., *Annona leptopetala* (R.E.Fr) H. Rainer, *Annona muricata* L., *Annona squamosa* L., *Annona cherimola* Mill., and Atemoya/Abdel Razek (hybrid of *A. squamosa* and *A. cherimola*) have been reported as cytotoxic agents [13,14,15,16,17,18,19,20]. On the other hand, the EO of *A. neoinsignis* has never been evaluated for its antitumor potential. In this work, we characterized the chemical composition of the EO from the leaves of *A. neoinsignis* and evaluated its anti-liver-cancer potential via in vitro and in vivo approaches.

## 2. Results and Discussion

### 2.1. Chemical Composition of the EO from the Leaves of A. neoinsignis

The chemical components of the EO extracted from the leaves of *A. neoinsignis* were determined and quantified via gas chromatograph connected to a mass spectrometer (GC–MS) and flame ionization detector (GC–FID). The volatile compounds were identified by their mass spectra and retention indices, which were then compared with previously published data. The oil had a greenish color and a yield of 0.18% in relation to the weight of the dry material and a density of 0.80 g/cm^3^. The EO is mainly composed of hydrocarbons of the monoterpene and sesquiterpene groups, as well as their derivatives. A total of 44 compounds were detected, representing 99.84% (Table 1).

The main compounds identified in the EO extracted from the leaves of *A. neoinsignis* were β-elemene (29.61%), (*E*)-caryophyllene (18.23%), germacrene D (15.34%), and germacrene B (6.80%), which together accounted for approximately 69.98% of the total identified oil (Figure 1 and Figures S1–S45). Other compounds identified at concentrations above 1.5% included bicyclogermacrene (4.61%), γ-elemene (4.47%), α-humulene (3.33%), α-copaene (2.52%), δ-elemene (2.27%), δ-amorphene (1.81%), and β-selinene (1.69%).

These findings corroborate what was reported in previous studies with EOs from Annonaceae species, particularly those belonging to the genus *Annona*. The prevalence of sesquiterpene hydrocarbons is common in the leaf EOs of plants of this family [23], and the main compounds found in the EO of *A. neoinsignis* resemble those identified in several species of the genus *Annona*, including the EOs extracted from the leaves of *A. coriacea* [24], *A. vepretorum* [25], and *A. squamosa* [26].

Furthermore, the occurrence of germacrene D, bicyclogermacrene, and (*E*)-caryophyllene as major compounds has also been detected in the chemical composition of the leaf EOs of *A. atemoya*, *A. senegalensis*, *A. pickelii*, *A. glabra,* and *A. foetida* [27]. Factors that can affect the chemical composition of EOs include temperature, seasonal changes, water supply, soil nutrients, circadian rhythm, and plant age [28].

### 2.2. Cytotoxicity of the EO Extracted from the Leaves of A. neoinsignis

The cytotoxicity of *A. neoinsignis* leaf EO was evaluated against six (HepG2, HCT116, MCF-7, MDA-MB-231, 4T1, and B16-F10) cancer cell lines and one (MRC-5) noncancerous cell line by the Alamar blue assay after 72 h of incubation (Figure S46). Table 2 shows the half maximum inhibitory concentration (IC_50_) values found. *A. neoinsignis* leaf EO had the lowest IC_50_ value (12.28 μg/mL) in the mouse melanoma cell line B16-F10, while the highest IC_50_ value (37.50 μg/mL) was found in the human breast cancer cell line MCF-7. Doxorubicin was used as a positive control, and the IC_50_ values ranged from 0.03 to 0.84 μg/mL for the liver cancer cell line HepG2 and the human breast cancer cell line MCF-7. For noncancer cell lines, *A. neoinsignis* leaf EO had IC_50_ values > 50 μg/mL for the human lung fibroblast line. Doxorubicin had an IC_50_ value of 1.96 μg/mL for the same cell line.

As mentioned previously, many EOs derived from *Annona* species have been shown to be cytotoxic. Among them, the cytotoxicity of EO extracted from *A. senegalensis* leaves was associated with the presence of caryophyllene oxide [13], whereas spathulenol was attributed, at least in part, to the cytotoxicity of EO extracted from *A. vepretorum* leaves [16]. In the EO from *A. neoinsignis* leaves, the main constituents are β-elemene, (*E*)-caryophyllene, germacrene D, and germacrene B. Importantly, these major components of *A. neoinsignis* leaf EO are known cytotoxic agents [29,30,31,32]. In any case, the cytotoxicity of this EO can be attributed to the combination of major and minor volatile compounds.

### 2.3. Induction of Apoptosis by A. neoinsignis Leaf EO in Liver Cancer Cells

**Figure 2:**
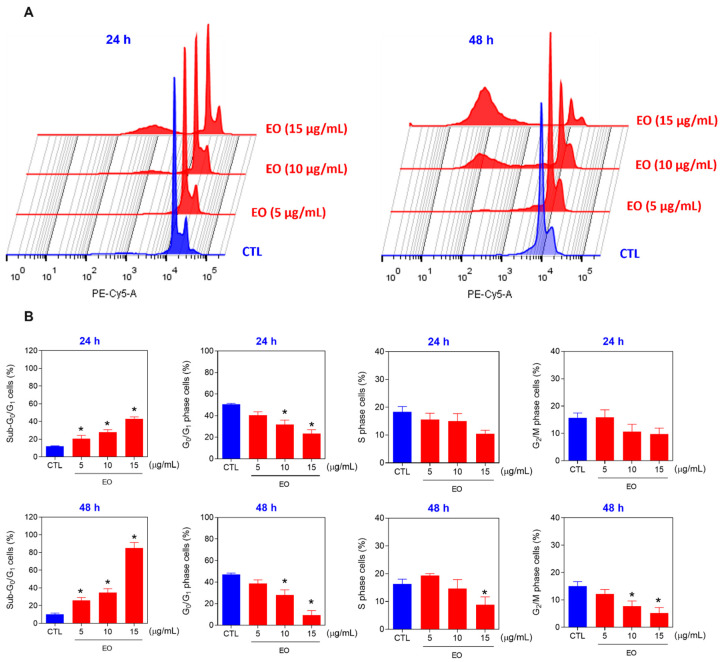
DNA fragmentation (sub-G_0_/G_1_ cells) and cell cycle phases (G_0_/G_1_, S, and G_2_/M) of HepG2 cells treated with *A. neoinsignis* leaf EO for 24 and 48 h. (**A**) Representative flow cytometric histograms. (**B**) Percentages of cells in sub-G_0_/G_1_, G_0_/G_1_, S, and G_2_/M. Vehicle (0.2% DMSO) was used as a negative control (CTL). The data are shown as the means ± S.E.M.s of three biological replicates performed in triplicate. * *p* < 0.05 compared with CTL by one-way ANOVA followed by Dunnett’s multiple comparisons test.

The liver cancer cell line HepG2 is sensitive to *A. neoinsignis* leaf EO and was selected for further experiments. Next, cell cycle analysis was also performed on HepG2 cells treated with EO from *A. neoinsignis* leaves at concentrations of 5, 10, and 15 μg/mL after 24 and 48 h of incubation (Figure 2). Propidium iodide (PI) staining was used to measure the DNA content of cells to identify sub-G_0_/G_1_, G_0_/G_1_, S, and G_2_/M cells via flow cytometry, and all cells with sub-G_0_/G_1_ (<2n) DNA content were considered to have fragmented DNA. Curiously, EO from *A. neoinsignis* leaves caused DNA fragmentation in HepG2 cells and proportionally reduced all phases of the cell cycle.

**Figure 3:**
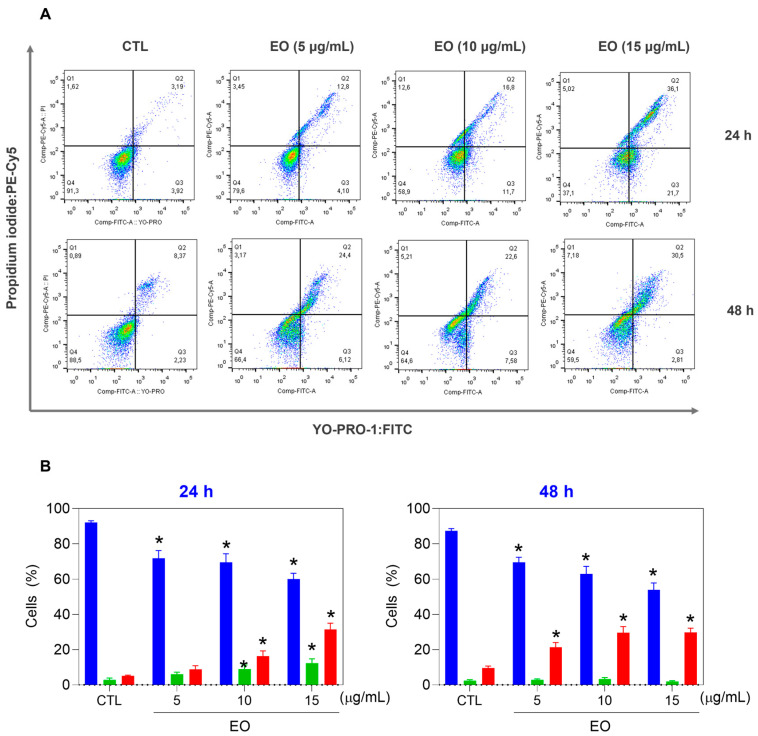
Effects of *A. neoinsignis* leaf EO on the induction of apoptosis in HepG2 cells after 24 and 48 h of treatment. (**A**) Representative flow cytometric dot plots. (**B**) Quantification of viable (YO-PRO-1/PI double-negative cells, blue bars), apoptotic (YO-PRO-1-positive/PI-negative cells, red bars), and dead (YO-PRO-1/PI double-positive cells plus YO-PRO-1-negative/PI-positive cells, green bars) cells. Vehicle (0.2% DMSO) was used as a negative control (CTL). The data are presented as the means ± S.E.M.s of three biological replicates performed in triplicate. * *p* < 0.05 compared with CTL by one-way ANOVA followed by Dunnett’s multiple comparisons test.

We then detected the viability of HepG2 cells treated with EO from *A. neoinsignis* leaves at concentrations of 5, 10, and 15 μg/mL via YO-PRO-1/PI double-staining after 24 and 48 h of incubation (Figure 3). For this purpose, viable cells (YO-PRO-1/PI double-negative cells), apoptotic cells (YO-PRO-1-positive/PI-negative cells), and dead cells (YO-PRO-1/PI double-positive cells plus YO-PRO-1-negative/PI-positive cells) were quantified via flow cytometry. In this case, dead cells indicate cells with unidentified types of cell death. Interestingly, *A. neoinsignis* leaf EO decreased the number of viable cells while increasing the number of apoptotic cells, followed by dead cells.

**Figure 4:**
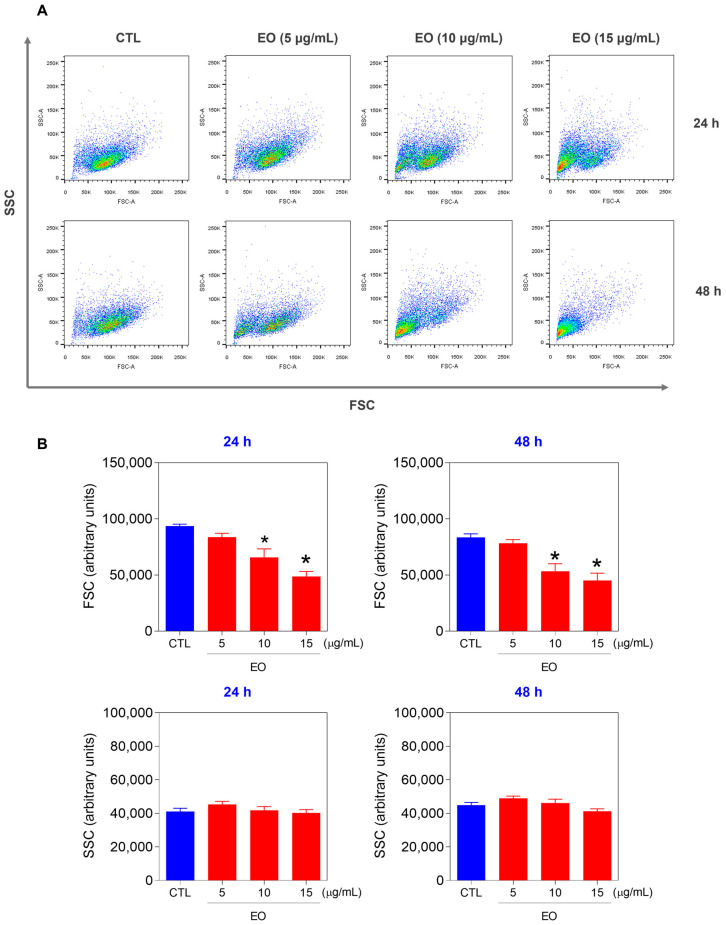
Effect of *A. neoinsignis* leaf EO on the cell size and granularity/complexity of HepG2 cells, as assessed by light scattering characteristics (FSC: forward scatter; SSC: side scatter) detected by flow cytometry after 24 and 48 treatments. (**A**) Representative flow cytometric dot plots. (**B**) Quantification of FSC and SSC. Vehicle (0.2% DMSO) was used as a negative control (CTL). The data are presented as the means ± S.E.M.s of three biological replicates performed in triplicate. * *p* < 0.05 compared with CTL by one-way ANOVA followed by Dunnett’s multiple comparisons test.

**Figure 5:**
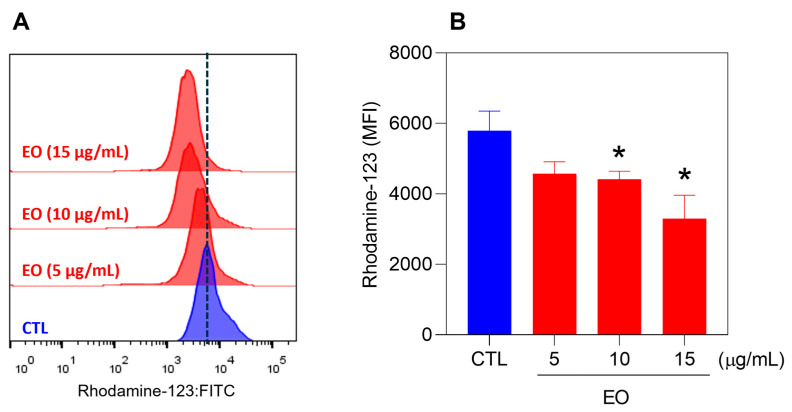
Mitochondrial depolarization in HepG2 cells treated with EO from *A. neoinsignis* leaves for 24 h. Mitochondrial potential was assessed with rhodamine-123-stained cells via flow cytometry. Vehicle (0.5% DMSO) was used as a negative control (CTL). The data are presented as the means ± S.E.M.s of three biological replicates performed in triplicate. * *p* < 0.05 compared with CTL by one-way ANOVA followed by Dunnett’s multiple comparisons test. MFI = Mean fluorescence intensity.

The cell size and granularity/complexity were also determined via flow cytometry via forward scatter (FSC) and side scatter (SSC), respectively. Treatment of HepG2 cells with EO from *A. neoinsignis* leaves reduced the FSC (Figure 4) after 24 and 48 h of incubation, indicating a reduction in cell size and corroborating cell death by apoptosis. Furthermore, the mitochondrial transmembrane potential was also measured via rhodamine-123 staining in HepG2 cells treated with EO from *A. neoinsignis* leaves at concentrations of 5, 10, and 15 μg/mL after 24 h of incubation. A significant reduction in the mitochondrial transmembrane potential was also detected in HepG2 cells treated with EO from *A. neoinsignis* (Figure 5), confirming that this EO can cause apoptosis in liver cancer cells.

Apoptosis is a type of regulated cell death characterized by a series of unique morphological and biochemical changes, such as decreased cell size, blebbing, DNA fragmentation, phosphatidylserine externalization, loss of the mitochondrial membrane potential, and caspase activation [33,34,35]. In this study, we found that *A. neoinsignis* leaf EO induced apoptosis in liver cancer cells, as evidenced by DNA fragmentation, loss of mitochondrial transmembrane potential, and YO-PRO-1/PI double-staining. Previously, Bomfim et al. [16] reported that EO from *A. vepretorum* leaves causes apoptosis in B16-F10 melanoma cells, as observed by the induction of phosphatidylserine externalization without affecting cell membrane integrity. Similarly, EO extracted from *A. squamosa* pericarp also caused apoptosis in SMMC-7721 liver cancer cells, as indicated by shrinkage or fragmentation of the cell nucleus [17].

β-Elemene and (*E*)-caryophyllene, two of the main constituents of *A. neoinsignis* leaf EO, have also been previously reported as inducers of apoptosis. β-Elemene caused G_2_/M cell cycle arrest and apoptotic cell death in non-small cell lung cancer cells, as observed by the induction of caspase-3, -7, and -9 activities; cytochrome c release; decreased Bcl-2 expression; and increased levels of DNA fragmentation [36]. β-Elemene also promoted apoptosis in colorectal cancer cells, as observed by nuclear chromatin condensation and phosphatidylserine externalization, decreased mitochondrial membrane potential, and cleavage of the caspase-3/9 and PARP proteins [37]. (*E*)-Caryophyllene induced apoptosis in liver cancer cells, as observed by the externalization of phosphatidylserine and the cleavage of caspase-3 and PARP [38].

### 2.4. Effect of A. neoinsignis Leaf EO on Mice Xenografted with Liver Cancer Cells

**Figure 6:**
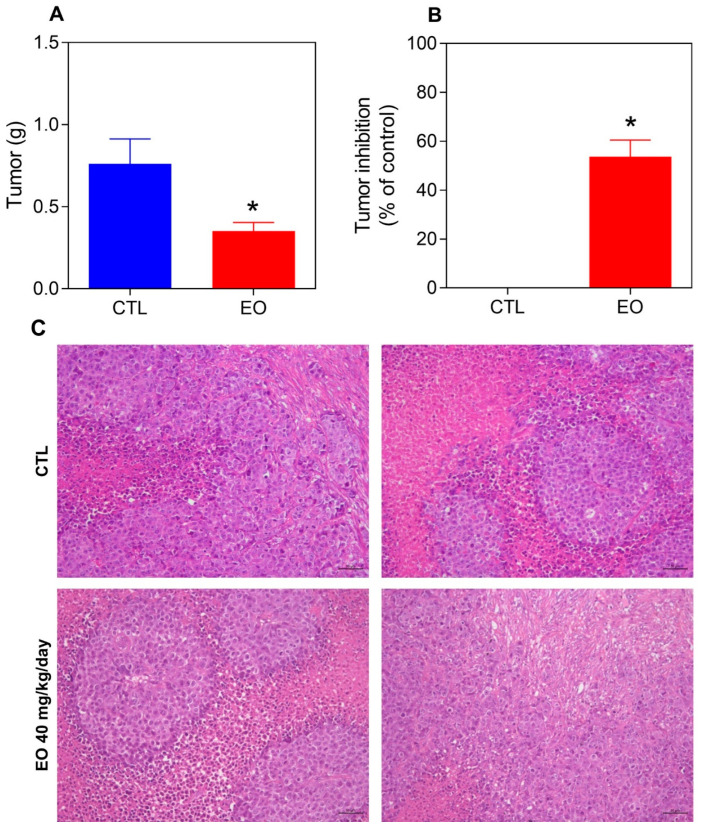
Effects of treatment with *A. neoinsignis* leaf EO on tumor weight (**A**) and tumor inhibition (**B**) in C.B.17 SCID mice bearing HepG2 cell xenografts. (**C**) Representative photomicrographs of HepG2 tumors. The treatments (40 mg/kg EO) were injected intraperitoneally into the mice daily for two weeks. Vehicle (5% DMSO) was used as a negative control (CTL). The data are presented as the means ± S.E.M.s of 8 animals. * *p* < 0.05 compared with CTL by two-tailed unpaired Student’s *t* test.

The in vivo antitumor effect of *A. neoinsignis* leaf EO was measured in C.B.17 SCID mice bearing HepG2 xenografts. One day after tumor inoculation, the animals were treated daily for two weeks with vehicle (5% DMSO, control group) or *A. neoinsignis* leaf EO at a dosage of 40 mg/kg. At the end of treatment, the mean tumor weight in the control group was 0.76 ± 0.15 g, whereas in the mice treated with EO, the mean tumor weight was 0.35 ± 0.05 g (Figure 6A), indicating a significant decrease of 53.7% (Figure 6B). These data indicate that *A. neoinsignis* leaf EO can effectively inhibit tumor growth in vivo.

Histological analysis revealed tumors with morphologies consistent with moderately differentiated hepatocellular carcinoma, delineated by a connective tissue capsule. The intratumor extracellular matrix is predominantly composed of collagen fibers, with sparse vascularization. Extensive areas of coagulative necrosis were observed throughout the tumor, generally accompanied by inflammatory cell infiltration in the peripheral regions. In certain areas, neoplastic cells are arranged in clusters resembling islands surrounded by a collagenous matrix or located near necrotic zones. Additionally, foci of invasion into adipose and cartilaginous tissues were identified. Tumors in the EO group presented granulation tissue adjacent to the necrotic areas (Figure 6C).

All the animals were weighed at the beginning and end of the experiment to assess weight gain or loss. The wet weights of the liver, heart, lungs, and kidneys were also determined to assess the toxicological potential of *A. neoinsignis* leaf EO. Interestingly, no significant changes in animal or organ weight were detected in the mice treated with *A. neoinsignis* leaf EO for two weeks at a dosage of 40 mg/kg (Figure S47).

The systemic toxicity of the EO was assessed through histological analysis of various organs. The tissue architecture of the heart and kidneys remained preserved in both groups, with only mild vascular hyperemia observed. In contrast, the hepatic parenchyma was partially preserved, with moderate hydropic degeneration of hepatocytes. Vascular hyperemia was also noted in the central venules and vessels of the portal triad, accompanied by inflammatory cell infiltration in these regions. The pulmonary parenchyma exhibited significant alterations, including areas of atelectasis resulting from thickening of the alveolar septa caused by hyperplasia and hypertrophy of pneumocytes, as well as hyperemia of the pulmonary capillaries. Additionally, focal areas of necrosis were identified in the bronchial epithelium, along with moderate active hyperemia and focal hemorrhages in the lung tissue (Figure S48).

In previous studies, *A. vepretorum* leaf EO, when microencapsulated with β-cyclodextrin and administered intraperitoneally at a dose of 50 mg/kg for 11 consecutive days, reduced the growth of B16-F10 melanoma cells in C57BL/6 mice by 62.66%, with no systemic toxicity detected [16]. Similarly, *A. leptopetala* EO inhibited sarcoma 180 tumor growth in Swiss mice by 59.29% and 58.77% after 7 days of intraperitoneal treatment at doses of 100 and 150 mg/kg, respectively. Moderate gastrointestinal toxicity was observed, but no genotoxicity was found at a dose of 350 mg/kg [18].

In conclusion, *A. neoinsignis* leaf EO demonstrated potent cytotoxicity to cancer cells, with liver cancer cells being sensitive. Furthermore, this EO caused apoptotic cell death in liver cancer cells and had the ability to reduce tumor growth in a human liver cancer xenograft model. These data suggest that *A. neoinsignis* leaf EO is an important source of anti-liver cancer drugs. Additionally, these results are described for the first time in *A. neoinsignis*, which suggests the need for further studies on its chemical and cytotoxic activity against tumor cell lines, as well as other biological activities, including mechanism of action and safety studies.

## 3. Materials and Methods

### 3.1. Botanical Material

The leaves of *A. neoinsignis* were collected in July 2024 at coordinates 3°05′51.2″ S and 59°58′34.7″ W on the campus of the Federal University of Amazonas (UFAM) in the metropolitan region of Manaus, Amazonas, Brazil. The species was identified by the biologist Deisy Pereira Saraiva from UFAM. An exsiccate of the species was deposited in the herbarium of the Department of Biology of UFAM (HUAM) under number 12,577. The accession received registration number A6E79F4 from the National System for the Management of Genetic Heritage and Associated Traditional Knowledge (SISGEN, Brazil).

### 3.2. EO Extraction

The leaves of *A. neoinsignis* were dried for 24 h in an oven with air circulation at 40 °C and then ground in a four-blade mill. The EO was extracted via the hydrodistillation method via a Clevenger system coupled to a 4 L round-bottom flask and boiled at 100 °C. The extractions were performed in triplicate, with 300 g of plant material added to each one, followed by distilled water to half the level of the flask. The EO was extracted for 3 h. Then, the oil was collected and filtered with anhydrous sodium sulfate to eliminate residual water and then stored in an amber bottle under refrigeration to avoid loss or degradation of chemicals. The oil yield, expressed as a percentage, was calculated via the following formula: Oil yield (%) = (mass of oil/mass of plant material) × 100.

### 3.3. Analysis of the Chemical Constituents

EO analysis was performed with a TRACE GC ULTRA/ISQ gas chromatograph (Thermo Scientific, Waltham, MA, USA) connected to an ISQ mass spectrometer equipped with a Tri Plus RSH autosampler (GC–MS) and flame ionization detector (GC–FID). A DB-5MS fused silica capillary column (film thickness of 30 m × 0.25 mm × 0.25 μm) coated with 5% phenylarylene-95% dimethylpolysiloxane was used for compound separation. Helium was employed as the retention gas, with a flow rate of 1.0 mL/min. The initial temperature was set at 40 °C for 4 min, followed by a flow rate of 4 °C/min up to 240 °C, then 10 °C/min up to 280 °C, and finally 280 °C for 2 min. The injector temperature was 250 °C, while the detector temperature was 280 °C [39]. The samples were produced by dissolving 10 mg in 1 mL of HPLC-grade ethyl acetate and injecting 1 μL of the solution in split mode at a ratio of 1:25. A typical solution of *n*-alkanes (C_8_–C_20_) was used to establish the retention indices, and calculations were performed via the van den Dool and Kratz equation [21]. The peak areas and retention times agreed electronically with an integrator. The stationary phase for GC–MS analysis was a DB-5MS fused silica capillary column (30 m × 0.25 mm × 0.25 μm film thickness) coated with 5% phenylarylene-95% dimethylpolysiloxane. Mass spectra were obtained at 70 eV with 0.5 s scan intervals and fragments ranging in size from 40 to 550 Da. Other analysis settings were similar to those used for the GC–FID analysis.

To identify the volatile constituents, the collected mass spectra were compared with those available in the literature [22], and retention indices were used. The proportion of each substance was calculated by dividing its area by the total area of all the substances in the sample and multiplying by 100.

### 3.4. Cytotoxicity Assay

The cancer cell lines HepG2 (human liver cancer), HCT116 (human colon cancer), MCF-7 (human breast cancer), MDA-MB-231 (human breast cancer), 4T1 (mouse breast cancer), and B16-F10 (mouse melanoma), together with the noncancerous cell line MRC-5 (human lung fibroblast), were used in this study. All cell lines were obtained from the American Type Culture Collection (ATCC; Manassas, VA, USA) and maintained following the ATCC animal cell culture guidelines [40]. To confirm the use of mycoplasma-free cells, all the cells were tested for mycoplasma with a mycoplasma staining kit (Sigma–Aldrich, St. Louis, MO, USA).

The Alamar blue assay was used to quantify the cell viability as described previously [41]. The cells were cultured in 96-well plates for each experiment at a concentration of 3 × 10^4^ cells/well for nonadherent cells or 7 × 10^3^ cells/well for adherent cells. EO was dissolved in pure dimethyl sulfoxide (DMSO, Vetec Química Fina Ltda., Duque de Caxias, RJ, Brazil), diluted in culture medium, and subsequently added (at eight different concentrations) to each well prior to a 72 h incubation. The DMSO concentration did not exceed 0.5%. Doxorubicin (IMA S.A.I.C., Buenos Aires, Argentina) served as a positive control, while untreated cells served as negative control. At the end of the treatment, 20 μL of 1 mM resazurin (Sigma–Aldrich, St. Louis, MO, USA) was added to each well. A SpectraMax 190 microplate reader was used to measure the absorbance at 570 and 600 nm (Molecular Devices, Sunnyvale, CA, USA). The percentage of cell inhibition was normalized to that of the negative control group. The IC_50_ values with 95% confidence intervals were obtained via nonlinear regression.

### 3.5. DNA Fragmentation and Cell Cycle Distribution Analysis

Cells stained with PI were used to quantify the cellular DNA content and measure internucleosomal DNA fragmentation and the cell cycle distribution [42]. Briefly, the cells were collected in a permeabilization solution containing 100 μg/mL RNase, 2 μg/mL PI, 0.1% Triton X-100, and 0.1% sodium citrate (all from Sigma–Aldrich, St. Louis, MO, USA), and flow cytometry was used to assess cellular fluorescence. A minimum of 10^4^ events were acquired for each sample. A BD LSRFortessa cytometer was used in conjunction with BD FACSDiva (BD Biosciences, San Jose, CA, USA) and FlowJo 10 (FlowJo LLC; Ashland, OR, USA) software. Cell debris was excluded from the analysis, whereas single cells were selected via H-FSCs vs. A-FSCs and/or H-SSCs vs. A-SSCs.

### 3.6. Apoptosis Staining Assay

YO-PRO-1 (Sigma–Aldrich, St. Louis, MO, USA) and PI (BD Biosciences, San Jose, CA, USA) dyes were used to quantify the percentage of apoptotic cells in culture, where YO-PRO-1-positive/PI-negative cells were considered apoptotic cells (early stage) [43]. Briefly, the cells were stained with a solution containing 1.5 µM PI plus 0.1 µM YO-PRO-1, and the cellular fluorescence was assessed via flow cytometry as described above.

### 3.7. Mitochondrial Transmembrane Potential Analysis

Cells stained with rhodamine-123 dye were used to evaluate the mitochondrial transmembrane potential [44]. The cells were incubated with 1 μg/mL rhodamine-123 (Sigma–Aldrich, St. Louis, MO, USA) for 15 min at 37 °C in the dark and then rinsed, after which the cellular fluorescence was quantified via flow cytometry as described above.

### 3.8. Human Liver Cancer Xenograft Model

Sixteen male specific pathogen-free C. B-17 SCID mice (8 weeks old and weighing 20–25 g) were acquired and housed in the vivarium of Fiocruz-Bahia (Salvador, Bahia, Brazil). The animals were kept in polyacrylic cages, with a maximum of five mice per unit, receiving food and water ad libitum. The environment was controlled with a 12 h light–dark cycle, with the light phase starting at 7 am. All experimental procedures were previously approved by the institution’s animal ethics committee (protocol no. 01/2021).

To establish the human liver cancer xenograft model, HepG2 cells (10^7^ cells/500 µL/animal) were injected subcutaneously into the left frontal axilla of the mice. After a single day, the animals received intraperitoneal treatment (200 µL/animal) daily for two weeks. The animals were divided into two groups: 1. Animals that received injections of vehicle (5% DMSO solution) used to dilute the EO (*n* = 8); and 2. Animals that received injections of EO at 40 mg/kg (*n* = 8). One day after the end of treatment, an overdose of anesthetic (ketamine–xylazine) was administered to euthanize the animals, after which the tumors were removed and weighed. The inhibition ratio (percentage) was determined via the following formula: inhibition ratio (percentage) = [(A − B)/A] × 100, where A represents the mean tumor weight of the negative control and B represents the tumor weight of the treatment group.

To evaluate the toxicological effects, the mice were weighed at the beginning and end of the experiment. Throughout the experimental period, the animals were monitored for the presence of behavioral or physiological changes. After euthanasia, the liver, kidneys, lungs and heart were removed and examined for significant lesions, color changes and/or evidence of hemorrhage. Histological analyses of the tumors and organs were performed via light microscopy after they were stained with hematoxylin–eosin (H and E) and periodic acid–Schiff (PAS), the latter being applied to the liver and kidneys. The tissues were previously fixed in 4% formaldehyde solution. Histopathological evaluation was performed via light microscopy at magnifications of 4×, 100×, 200×, and 400×. The distributed parts were classified as absent (0), mild (+1), moderate (+2), and intense (+3). Representative photomicrographs were acquired via LAS v4 image acquisition software (LMD6500), adapted to the Leica specification.

### 3.9. Statistical Analysis

The data are presented as the means ± standard errors of the means (S.E.M.) or as IC_50_ values with 95% confidence intervals (95% CI) from at least three independent experiments performed in duplicate or triplicate. Two-tailed unpaired Student’s *t* test or one-way ANOVA followed by Dunnett’s test were used to compare differences among experimental groups (*p* < 0.05). GraphPad Prism version 8 was used to perform all the statistical analyses (Intuitive Software for Science; San Diego, CA, USA).

## Figures and Tables

**Figure 1:**
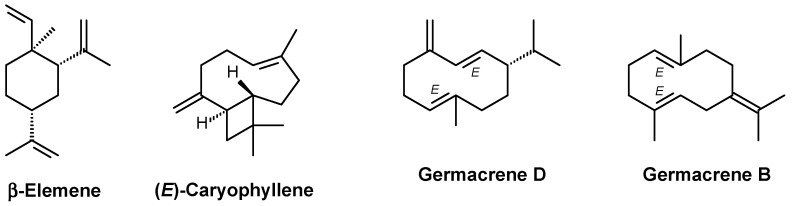
Main compounds identified in *A. neoinsignis* leaf EO.

**Table 1:** Chemical composition of the EO extracted from the leaves of *A. neoinsignis*.

Compounds		RI ^a^	RI ^b^	Peak Area %
*1*	β-Pinene	970	974	0.04 ± 0.00
*2*	Myrcene	988	988	0.11 ± 0.01
*3*	Limonene	1027	1024	0.07 ± 0.01
*4*	(*Z*)-β-Ocimene	1035	1032	0.04 ± 0.00
*5*	(*E*)-β-Ocimene	1046	1044	0.20 ± 0.02
*6*	Terpinolene	1083	1086	0.02 ± 0.00
*7*	Linalool	1098	1095	0.39 ± 0.02
*8*	Terpinen-4-ol	1179	1174	0.05 ± 0.00
*9*	α-Terpineol	1193	1186	0.24 ± 0.05
*10*	Nerol	1223	1227	0.01 ± 0.00
*11*	Geraniol	1250	1249	0.04 ± 0.00
*12*	δ-Elemene	1334	1335	2.27 ± 0.20
*13*	α-Cubebene	1346	1348	0.64 ± 0.07
*14*	α-Ylangene	1367	1373	0.41 ± 0.10
*15*	α-Copaene	1374	1374	2.52 ± 0.25
*16*	β-Elemene	1389	1389	29.61 ± 3.80
*17*	(*E*)-Caryophyllene	1419	1417	18.23 ± 0.43
*18*	*γ*-Elemene	1428	1434	4.47 ± 0.04
*19*	α-*trans*-Bergamotene	1432	1432	0.87 ± 0.02
*20*	Aromadendrene	1437	1439	0.05 ± 0.00
*21*	*cis*-Muurola-3,5-diene	1448	1448	0.10 ± 0.00
*22*	α-Humulene	1454	1452	3.33 ± 0.06
*23*	*cis*-Cadina-1(6),4-diene	1461	1461	0.05 ± 0.00
*24*	*γ*-Muurolene	1473	1478	0.73 ± 0.02
*25*	Germacrene D	14.80	1480	15.34 ± 2.13
*26*	β-Selinene	1488	1489	1.69 ± 0.07
*27*	Bicyclogermacrene	1494	1500	4.61 ± 0.15
*28*	α-Muurolene	1497	1500	0.20 ± 0.01
*29*	Germacrene A	1506	1508	0.82 ± 0.08
*30*	*γ*-Cadinene	1511	1513	0.28 ± 0.00
*31*	δ-Amorphene	1516	1511	1.81 ± 0.06
*32*	*trans*-Calamenene	1520	1521	0.12 ± 0.04
*33*	(*E*)-*γ*-Bisabolene	1525	1529	0.57 ± 0.01
*34*	*trans*-Cadina-1(2),4-diene	1531	1533	0.21 ± 0.01
*35*	α-Cadinene	1535	1537	0.32 ± 0.01
*36*	Selina-3,7(11)-diene	1540	1545	0.15 ± 0.00
*37*	Germacrene B	1558	1559	6.80 ± 0.39
*38*	Spathulenol	1575	1577	0.28 ± 0.02
*39*	Caryophyllene oxide	1580	1582	0.65 ± 0.02
*40*	Globulol	1584	1590	0.10 ± 0.00
*41*	1-*epi*-Cubenol	1626	1627	0.33 ± 0.00
*42*	Cubenol	1642	1645	0.25 ± 0.01
*43*	α-Cadinol	1653	1652	0.27 ± 0.01
*44*	*neo*-Intermedeol	1657	1658	0.55 ± 0.01
Monoterpenes				1.22%
Sesquiterpenes				98.63%
Total Identified				99.84%
*Total Not Identified (N.I.)*				0.15%

Note: The data are presented as the means ± S.D.s of three analyses. RI ^a^ (experimental retention indices): this index was calculated on a TR-5MS capillary column (30 m × 0.25 mm × 0.25 µm) according to Van Den Dool and Kratz [21], which is based on a homologous series of normal alkanes. RI ^b^ (literature retention indices): according to Adams [22]. N.I. = Not identified.

**Table 2:** Cytotoxicity of *A. neoinsignis* leaf EO.

Cells	Histological Type	IC_50_ and 95% CI (in μg/mL)	
		DOX	EO
Cancer cells			
HepG2	Human liver cancer	0.03 0.02–0.04	27.90 20.44–36.66
HCT116	Human colon cancer	0.05 0.04–0.06	24.35 16.86–37.23
MCF-7	Human breast cancer	0.84 0.65–1.12	37.50 30.07–55.21
MDA-MB-231	Human breast cancer	0.61 0.47–0.82	31.78 24.42–44.90
4T1	Mouse breast cancer	0.60 0.46–0.81	21.64 17.05–27.32
B16-F10	Mouse melanoma	0.06 0.04–0.08	12.28 10.08–14.85
Noncancerous cells			
MRC-5	Human lung fibroblast	1.94 1.40–2.88	>50

Note: Doxorubicin (DOX) was used as a positive control.

## Data Availability

The data presented in this study are available in the Supplementary Materials.

## Supplementary Materials

The following supporting information can be downloaded at: https://www.mdpi.com/article/10.3390/molecules30142971/s1, Figure S1: chromatogram of the total ions; Figure S2–S45: Mass spectrum of compounds. Figure S46: Concentration–response curves; Figure S47: The body weight and relative organ weight; Figure S48: Representative photomicrographs of organs.
