# Supplementary material for: Essential Oil from the Leaves of Annona neoinsignis H. Rainer (Annonaceae) Against Liver Cancer: In Vitro and In Vivo Studies
Source: Molecules. 2025 Jul 15;30(14):2971. doi: 10.3390/molecules30142971 (PMC12297985; doi:10.3390/molecules30142971)
Supplement: Supplementary file 1 [file molecules-30-02971-s001.zip › molecules-3722904-supplementary.pdf]

Supplementary Material

Essential oil from the leaves of *Annona neoinsignis* H. Rainer (Annonaceae) against liver cancer: *in vitro* and *in vivo* studies

Melissa P. Souza[1,2,†], Maria V. L. de Castro[3,†], Gabriela A. da C. Barbosa[3], Sabrine G. Carvalho[3], Amanda M. R. M. Coelho[3], Rosane B. Dias[3,4], Milena B. P. Soares[3,5], Emmanoel V. Costa[1,2]*, Daniel P. Bezerra[3,*]

[1]Postgraduate Program in Chemistry, Institute of Exact Sciences, Federal University of Amazonas (UFAM), Manaus, Amazonas, 69080-900, Brazil.

[2]Department of Chemistry, Institute of Exact Sciences, Federal University of Amazonas (UFAM), Manaus, Amazonas, 69080-900, Brazil.

[3]Gonçalo Moniz Institute, Oswaldo Cruz Foundation (IGM-FIOCRUZ/BA), Salvador, Bahia, 40296-710, Brazil.

[4]Department of Biological Sciences, State University of Feira de Santana, Feira de Santana, Bahia, 44036-900, Brazil.

[5]SENAI Institute for Innovation in Advanced Health Systems, SENAI CIMATEC, Salvador, BA 41650-010, Brazil.

† These authors contributed equally to this work.

*Corresponding authors:

E. V. Costa (e-mail: emmanoelvc@gmail.com or evc@ufam.br); D. P. Bezerra (e-mail: daniel.bezerra@fiocruz.br); Tel./Fax: +55-92-3305-1181 Ramal 2870 (E.V. Costa); Tel./Fax: +55-71-3176-2272 (D. P. Bezerra).

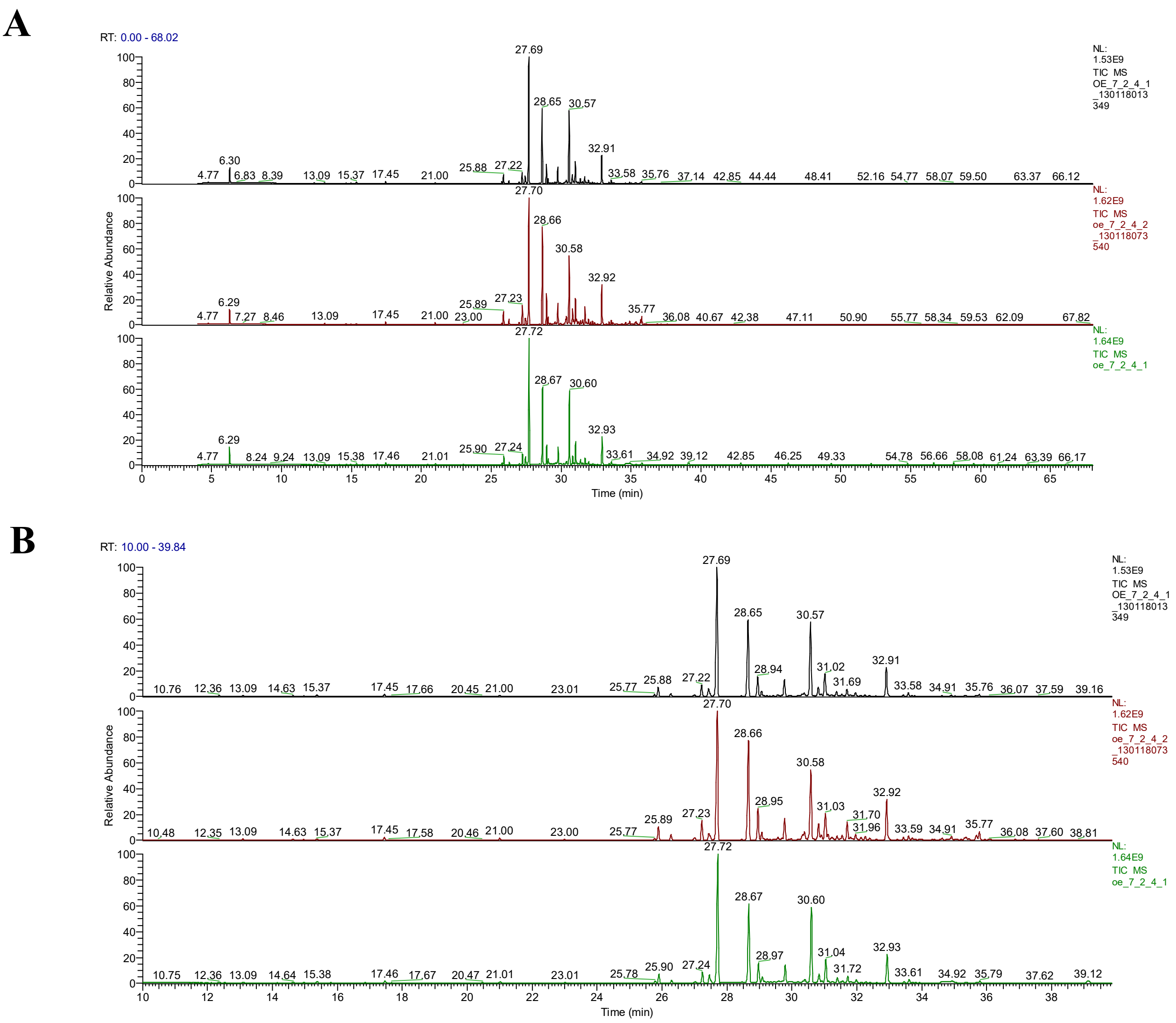

Figure S1. (**A**) Chromatogram of the total ions of *A. neoinsignis* leaf EO (triplicate); (**B**) Enlargement of the region between 10.0 min and 40.0 min.

Retention time of some main constituents present in the OE:

**β-pinene** (12.36 min)	**myrcene** (13.09 min)	**limonene** (14.63; 14.64 min)	**(*Z*)-β-ocimene** (14.96 min)
**(*E*)-β-ocimene** (15.37; 15.38 min)	**terpinolene** (16.85 min)	**linalool** (17.45; 17.46 min)	**terpinen-4-ol** (20.45; 20.46; 20.47 min)
**α-terpineol** (21.00; 21.01 min)	**nerol** (22.07 min)	**geraniol** (23.00; 23.01 min)	**δ-elemene** (25.88; 25.89; 25.90 min)
**α-cubebene** (26.27; 26.28; 26.30 min)	**α-ylangene** (27.00; 27.03 min)	**α-copaene** (27.22; 27.23; 27.24 min)	**β-elemene** (27.69; 27.70; 27.72 min)
**(*E*)-caryophyllene** (28.65; 28.66; 28.67 min)	**γ-elemene** (28.94; 28.95; 28.97 min)	**α-*trans*-bergamotene** (29.07; 29.09 min)	**aromadendrene** (29.24 min)
***cis*-muurola-3,5-diene** (29.57 min)	**α-humulene** (29.77; 29.79 min)	***cis*-cadina-1(6),4-diene** (29.98 min)	**γ-muurolene** (30.38 min)
**germacrene D** (30.57; 30.58; 30.60 min)	**β-selinene** (30.81; 30.82; 30.83 min)	**bicyclogermacrene** (31.02; 31.03; 31.04 min)	**α-muurolene** (31.10; 31.11; 31.13 min)
**germacrene A** (31.37; 31.39; 31.40 min)	**γ-cadinene** (31.54; 31.55; 31.56 min)	**δ-amorphene** (31.69; 31.70; 31.72 min)	***trans*-calamenene** (31.79; 31.82 min)
**(*E*)-γ-bisabolene** (31.95; 31.96; 31.98 min)	***trans*-cadina-1,4-diene** (32.12; 32.13; 32.14 min)	**α-cadinene** (32.25; 32.26; 32.28 min)	**selina-3,7(11)-diene** (32.39; 32.40; 32.41 min)
**germacrene B** (32.91; 32.92; 32.93 min)	**spathulenol** (33.43; 33.45 min)	**caryophyllene oxide** (33.58; 33.59; 33.61 min)	**globulol** (33.69; 33.70 min)
**1-*epi*-cubenol** (34.91; 34.92 min)	**cubenol** (35.35; 35.36 min)	**α-cadinol** (35.67; 35.68; 35.70 min)	**neo-intermedeol** (35.76; 35.77; 35.79 min)

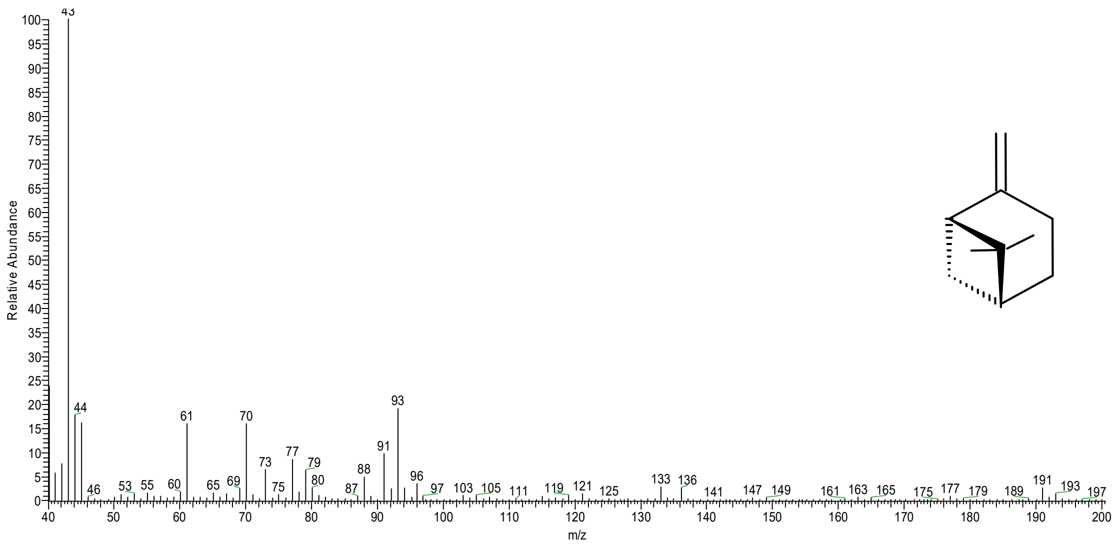

Figure S2. Mass spectrum of β-pinene (t_R 12.36 min).

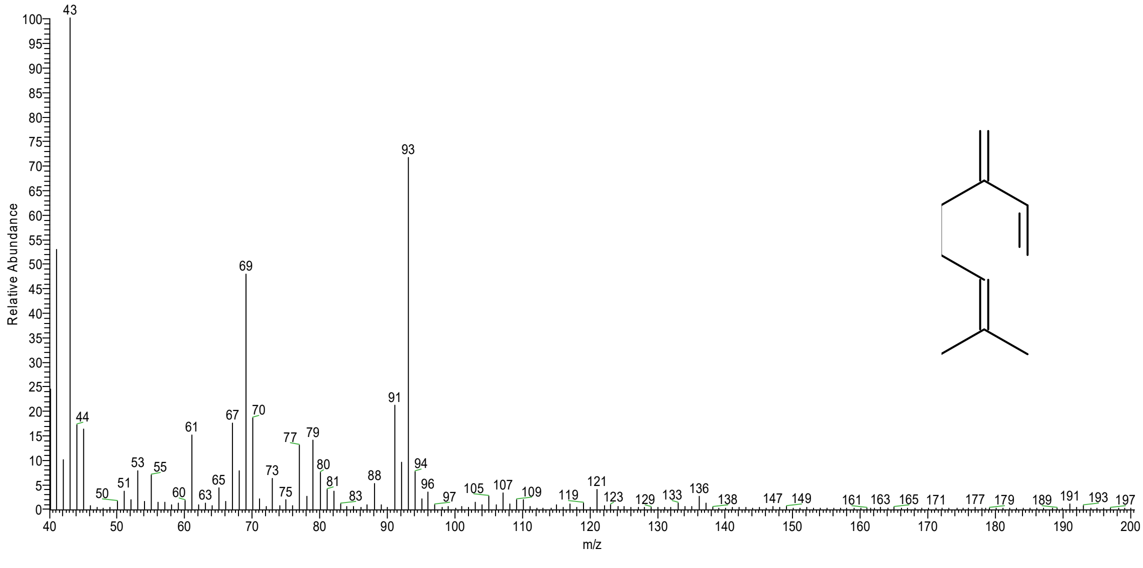

Figure S3. Mass spectrum of myrcene (t_R 13.09 min).

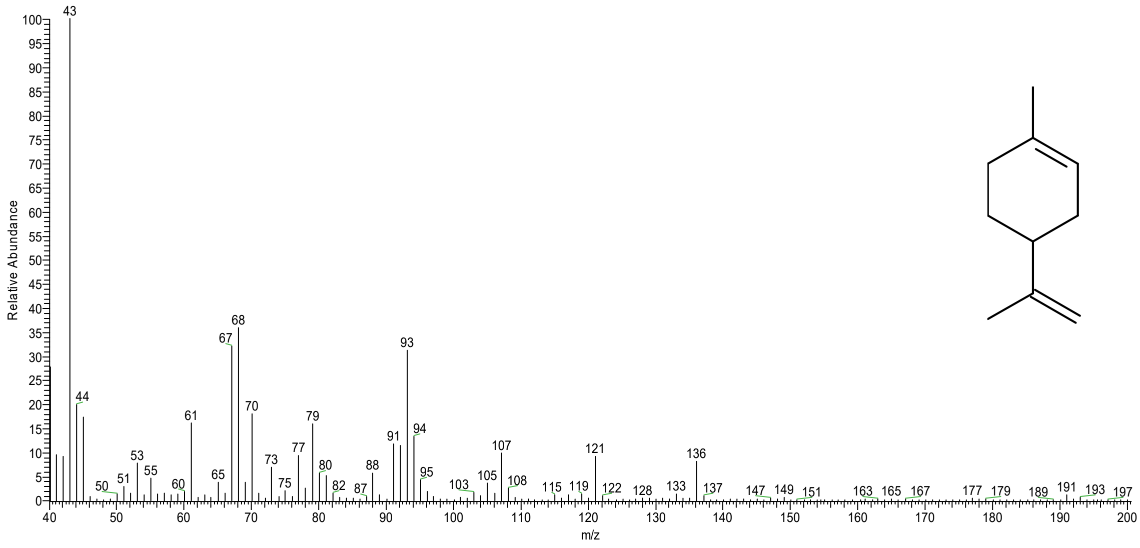

Figure S4. Mass spectrum of limonene (t_R 14.63; 14.64 min).

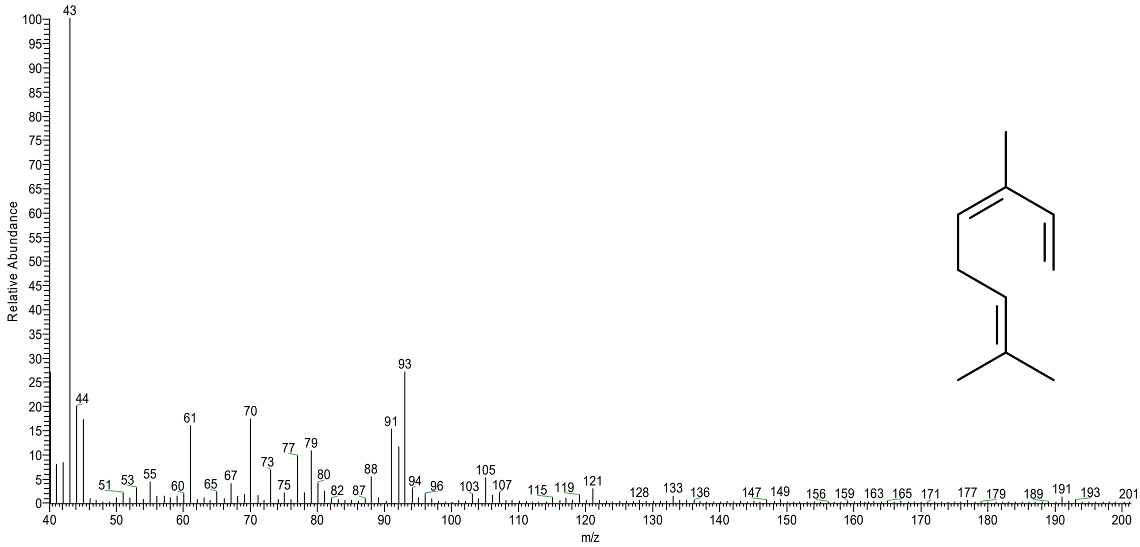

Figure S5. Mass spectrum of (*Z*)-β-ocimene (t_R 14.96 min).

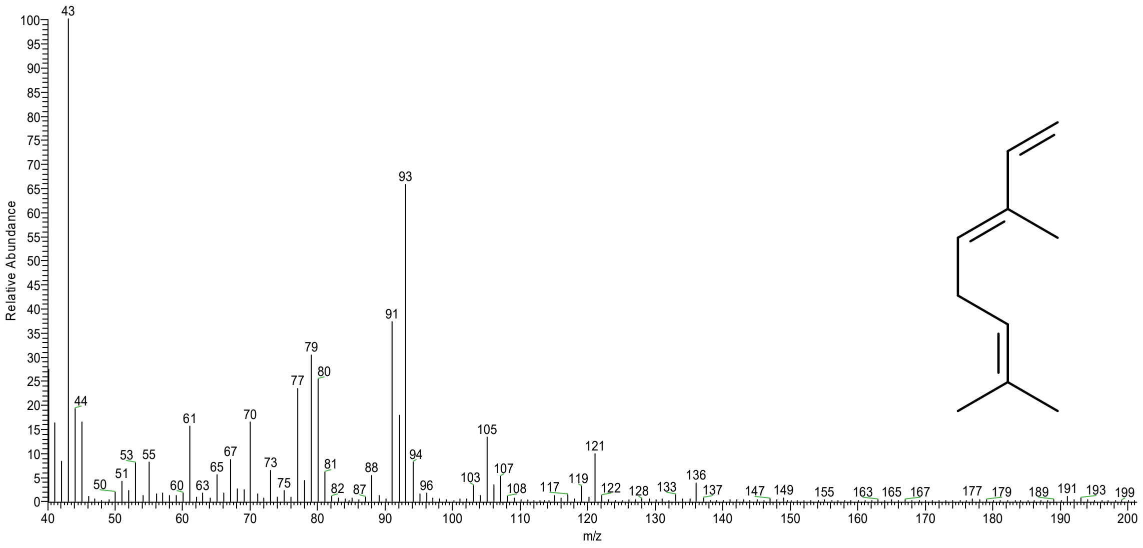

Figure S6. Mass spectrum of (*E*)-β-ocimene (t_R 15.37; 15.38 min).

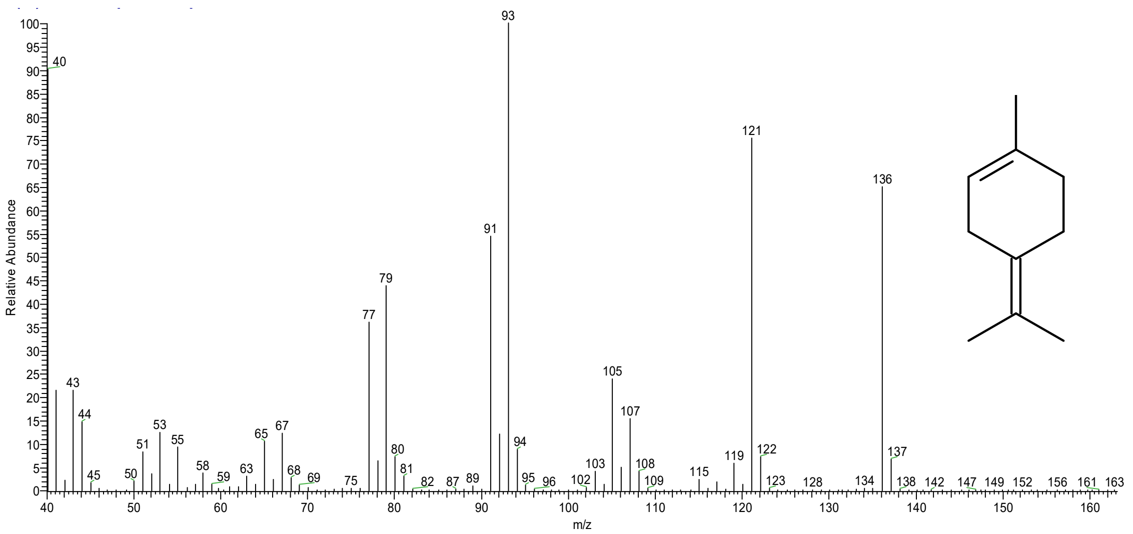

Figure S7. Mass spectrum of terpinolene (t_R 16.85 min).

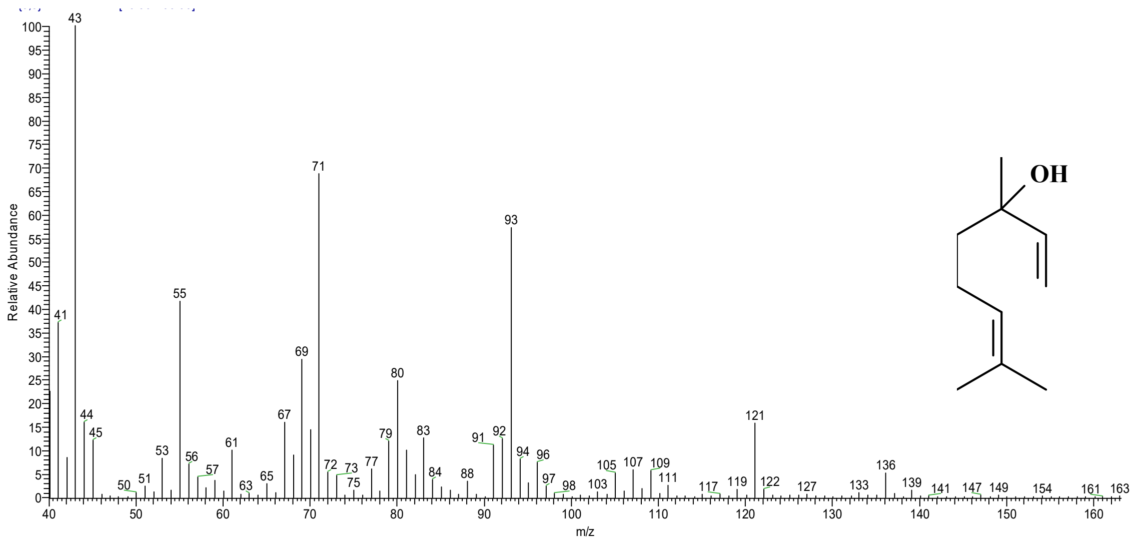

Figure S8. Mass spectrum of linalool (t_R 17.45; 17.46 min).

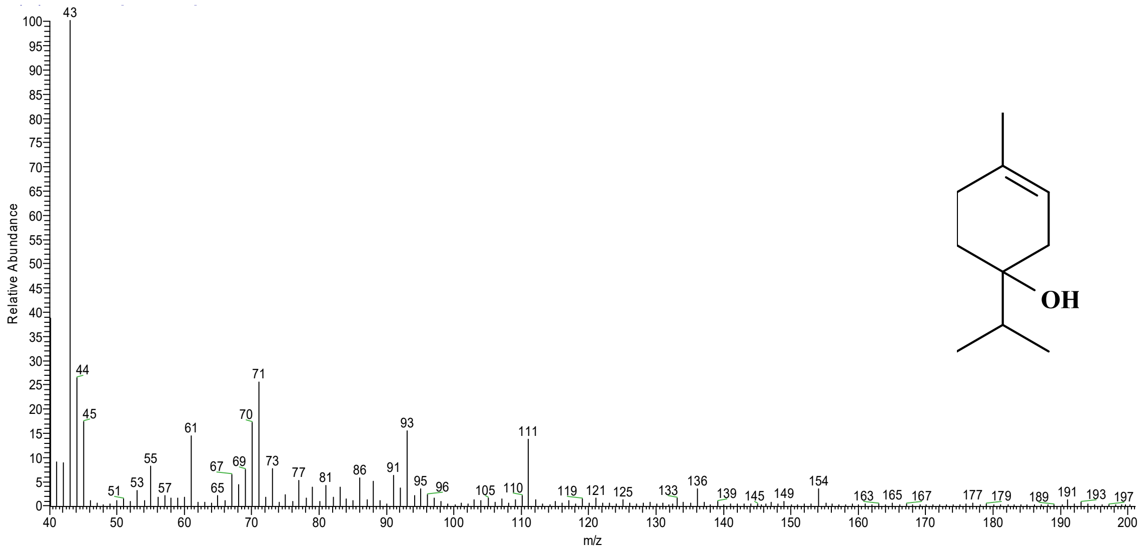

Figure S9. Mass spectrum of terpinen-4-ol (t_R 20.45; 20.46; 20.47 min).

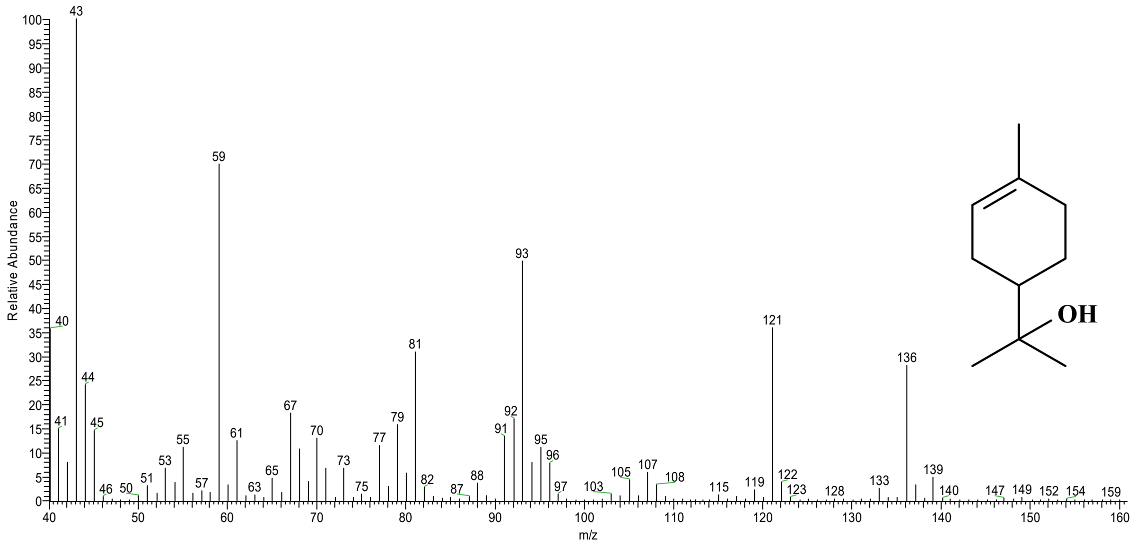

Figure S10. Mass spectrum of α-terpineol (t_R 21.00; 21.01 min).

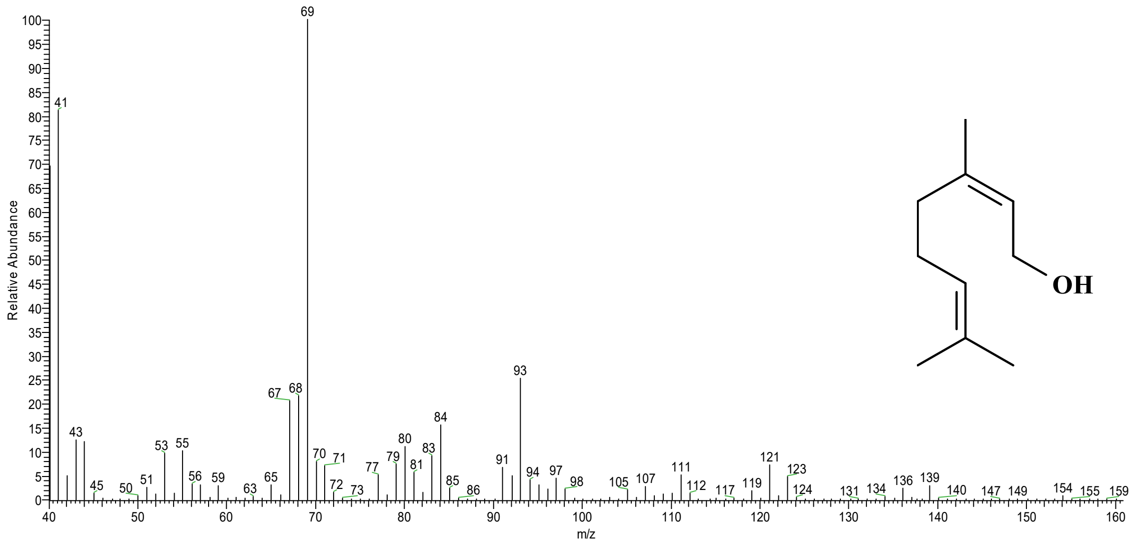

Figure S11. Mass spectrum of nerol (t_R 22.07 min).

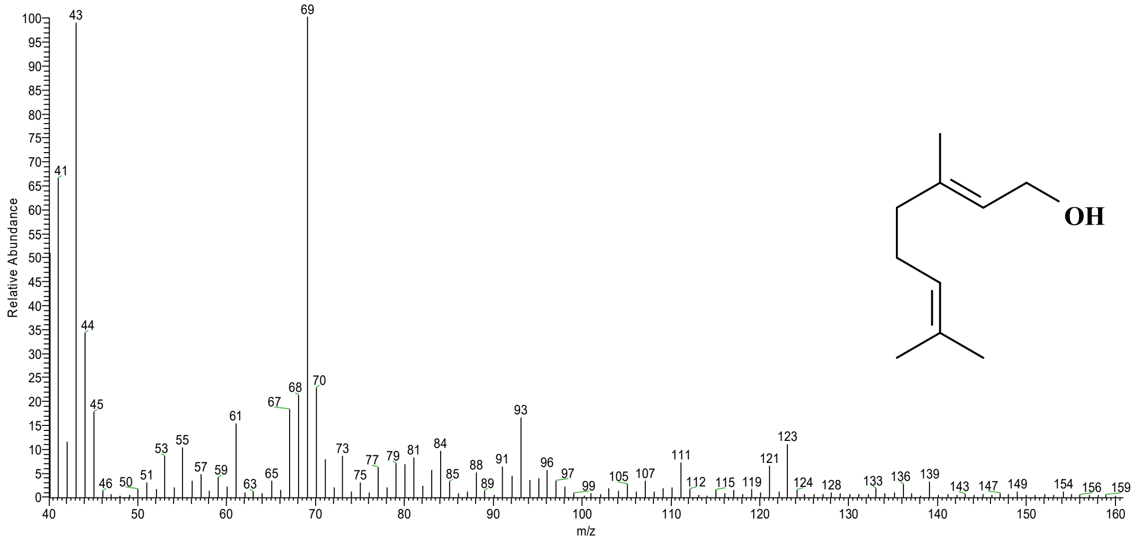

Figure S12. Mass spectrum of geraniol (t_R 23.00; 23.01 min).

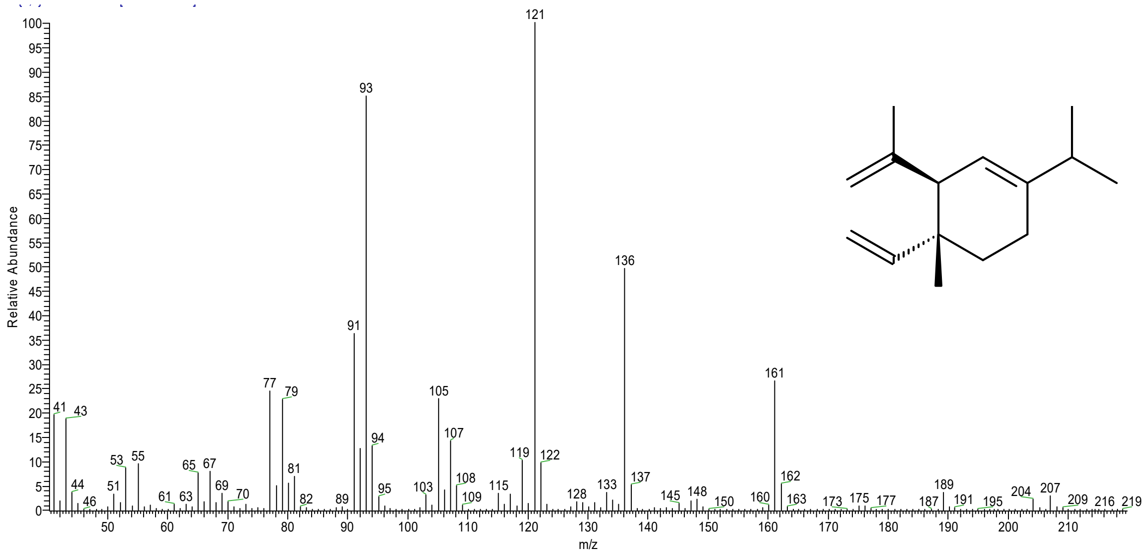

Figure S13. Mass spectrum of δ-elemene (t_R 25.88; 25.89; 25.90 min).

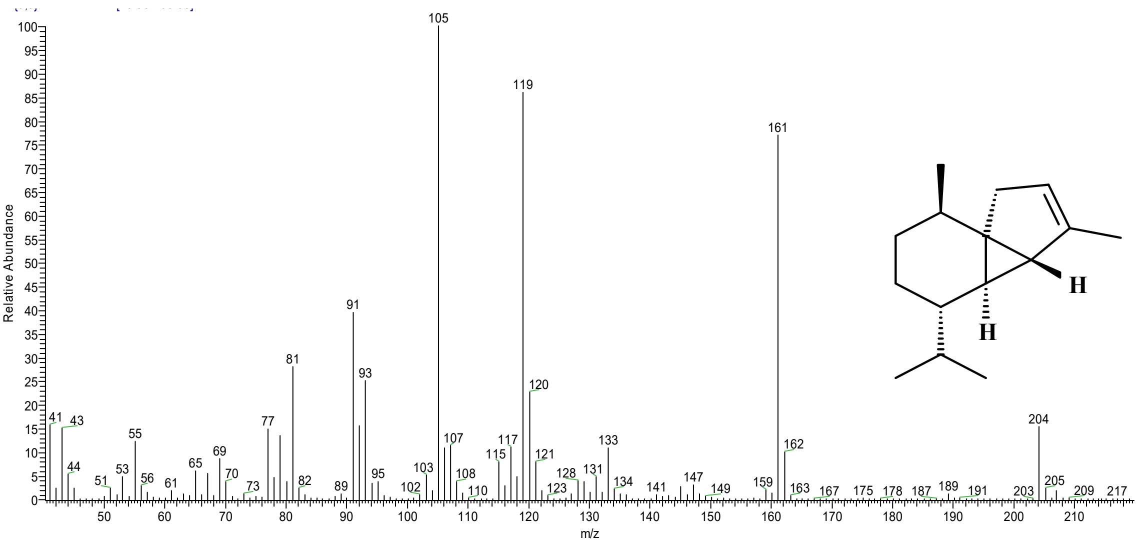

Figure S14. Mass spectrum of α-cubebene (t_R 26.27; 26.28; 26.30 min).

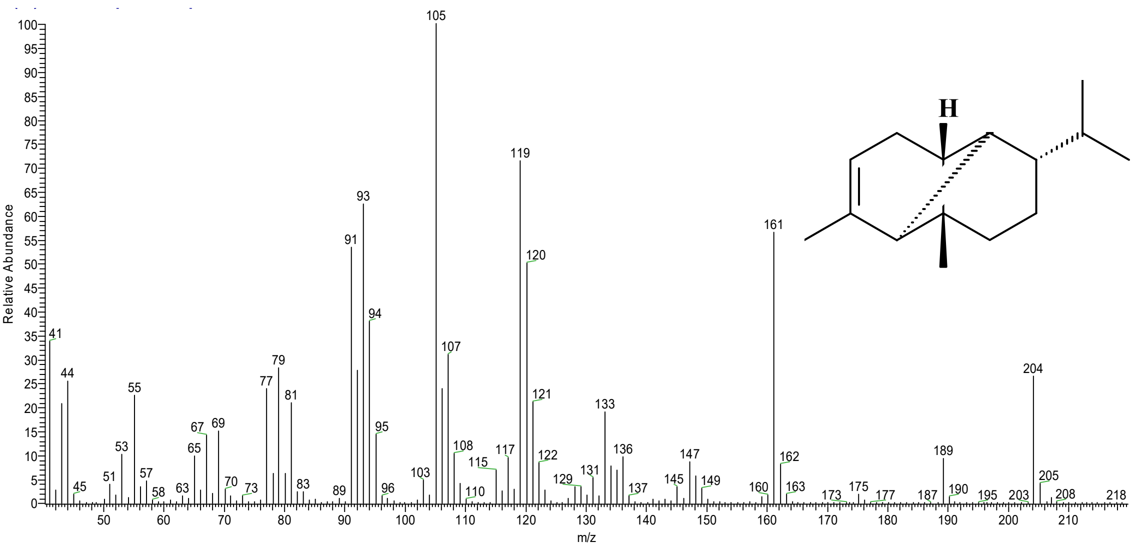

Figure S15. Mass spectrum of α-ylangene (t_R 27.00; 27.03 min).

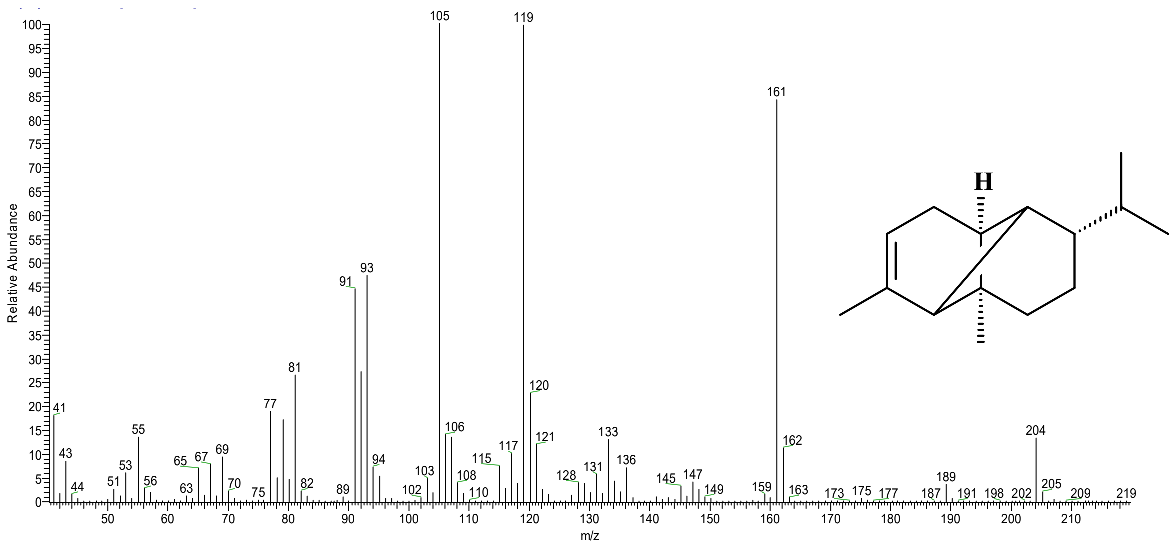

Figure S16. Mass spectrum of α-copaene (t_R 27.22; 27.23; 27.24 min).

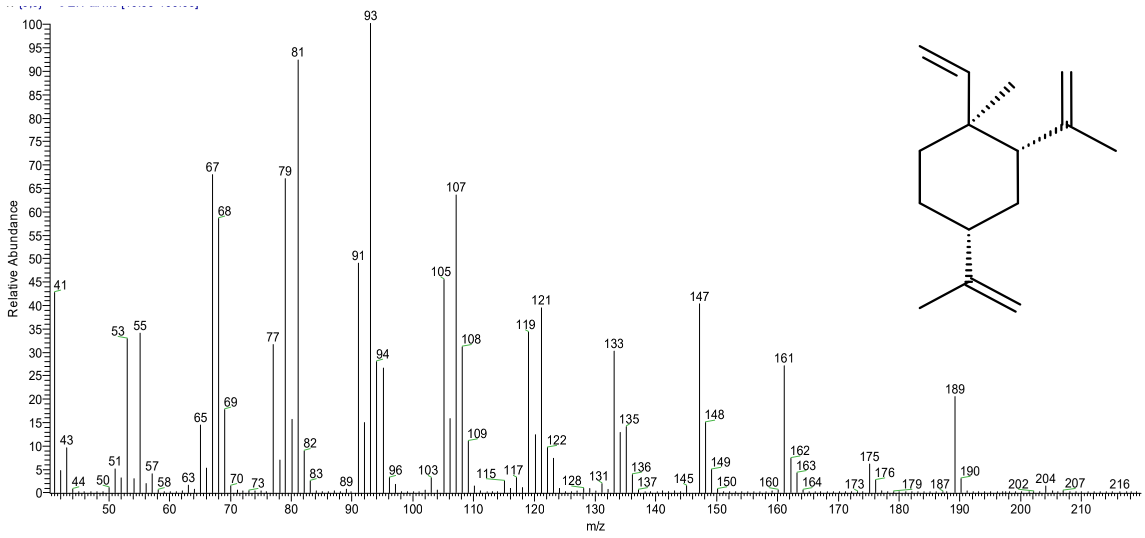

Figure S17. Mass spectrum of β-elemene (t_R 27.69; 27.70; 27.72 min).

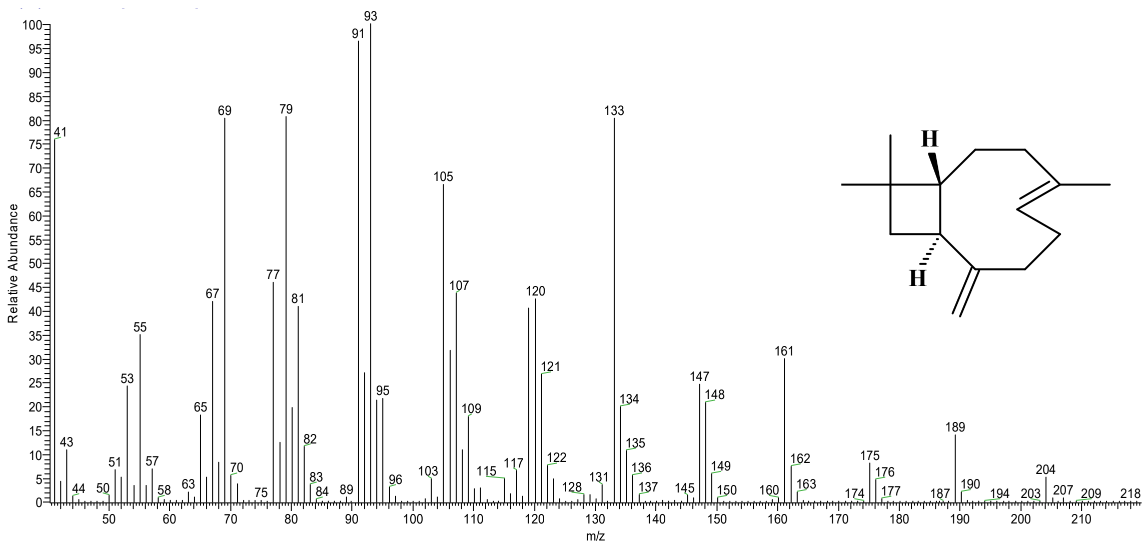

Figure S18. Mass spectrum of (*E*)-caryophyllene (t_R 28.65; 28.66; 28.67 min).

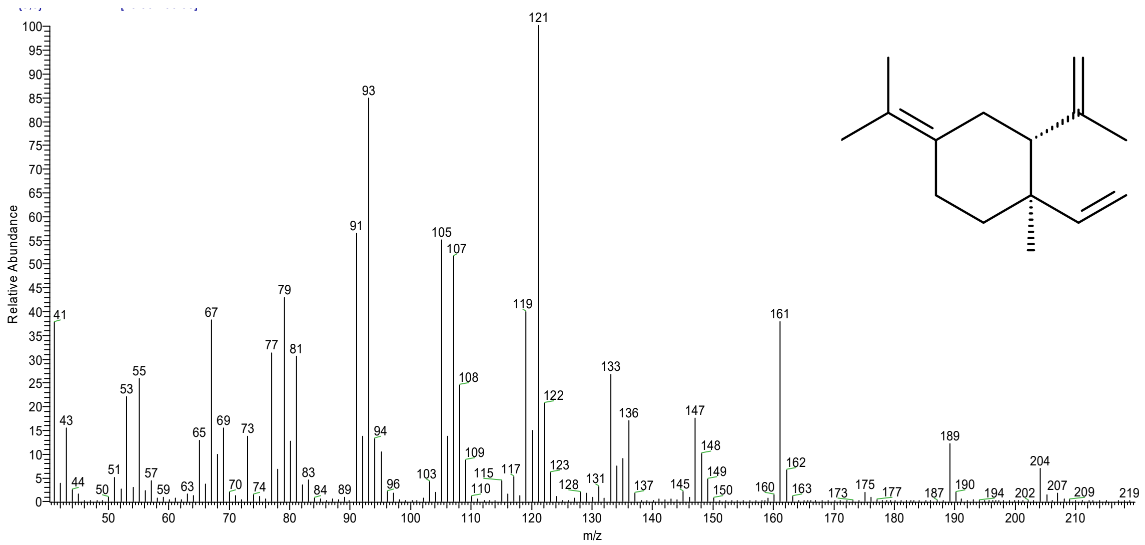

Figure S19. Mass spectrum of *γ*-elemene (t_R 28.94; 28.95; 28.97 min).

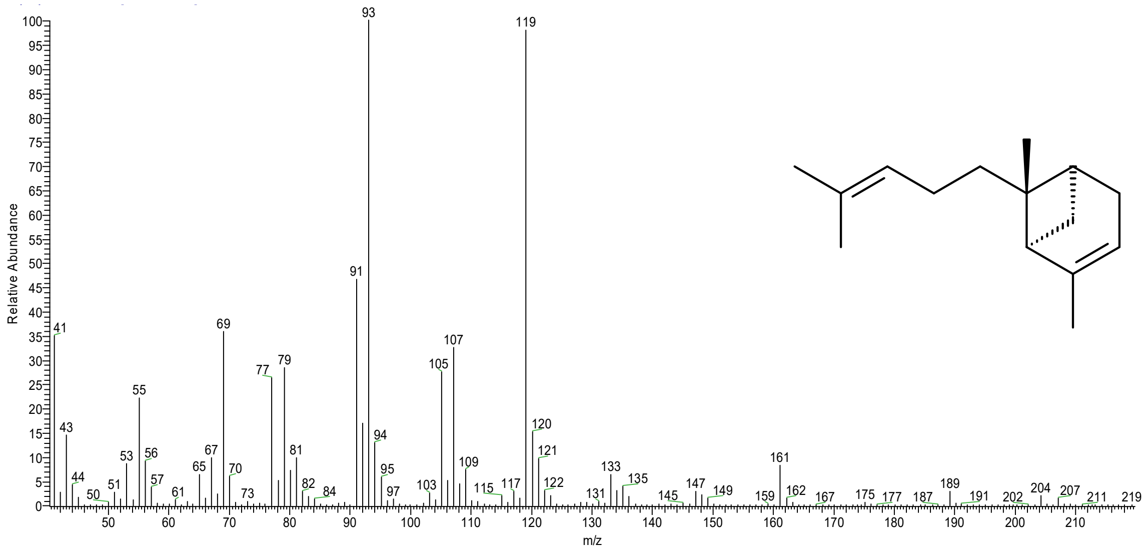

Figure S20. Mass spectrum of α-*trans*-bergamotene (t_R 29.07; 29.09 min).

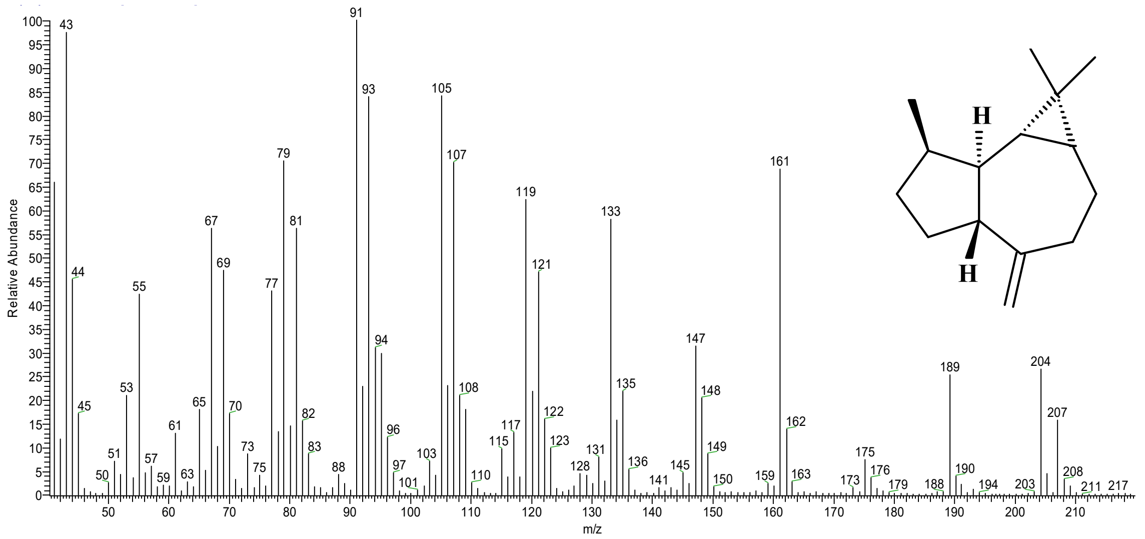

Figure S21. Mass spectrum of Aromadendrene (t_R 29.24 min).

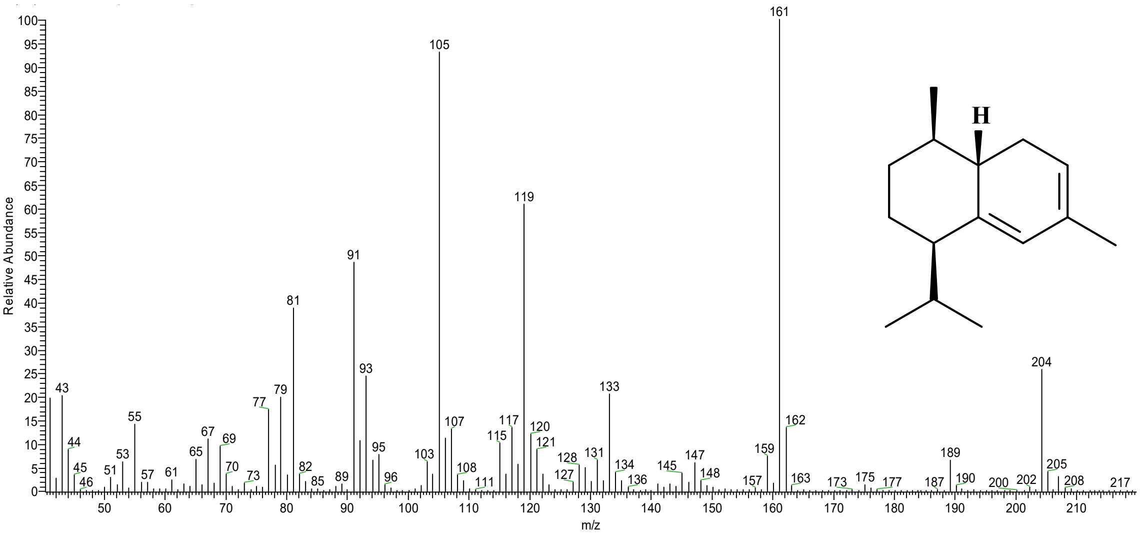

Figure S22. Mass spectrum of *cis*-muurola-3,5-diene (t_R 29.57 min).

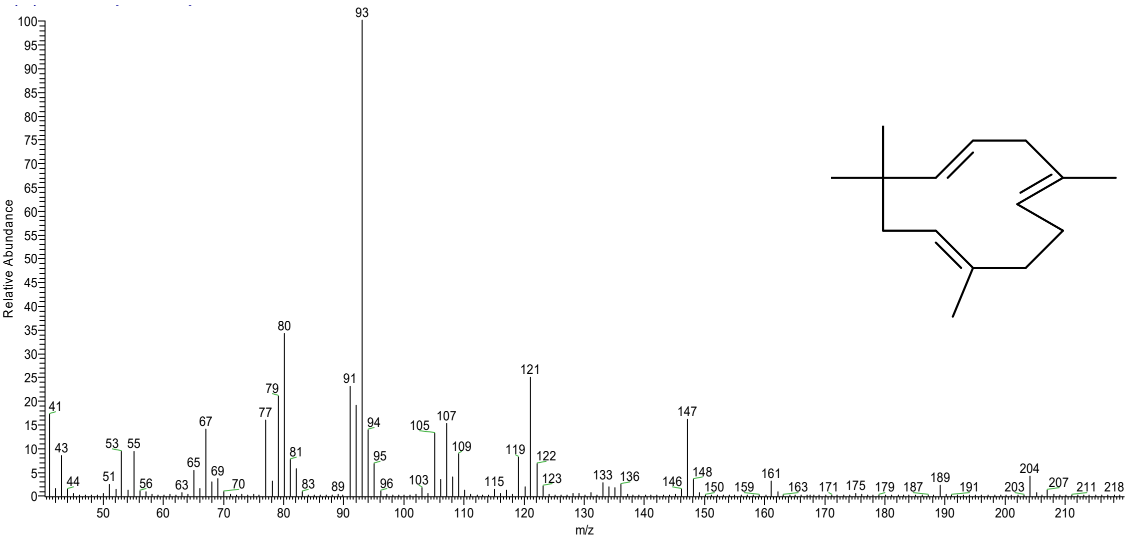

Figure S23. Mass spectrum of α-humulene (t_R 29.77; 29.79 min).

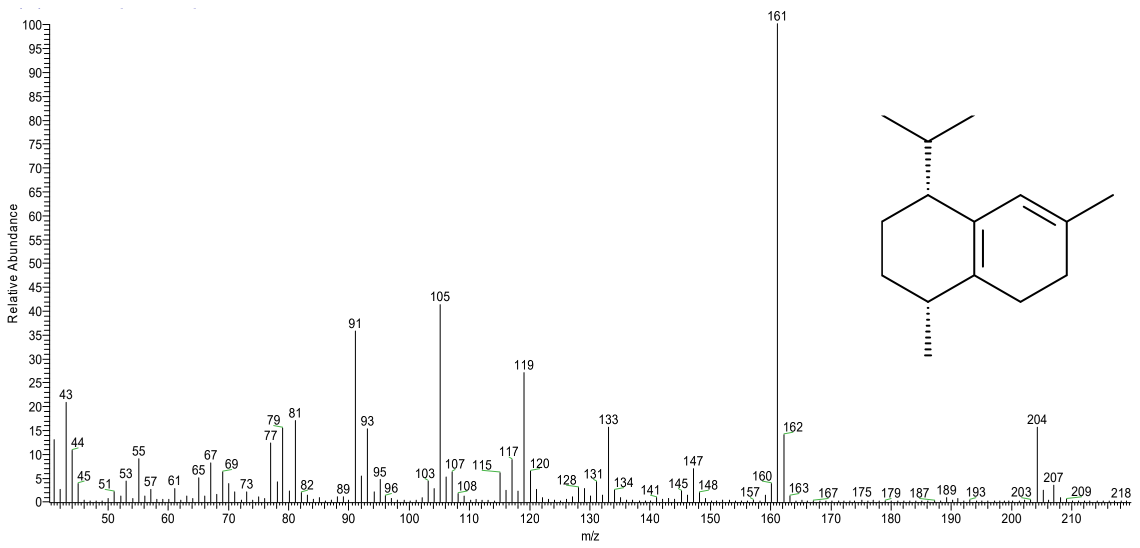

Figure S24. Mass spectrum of *cis*-cadina-1(6),4-diene (t_R 29.98 min).

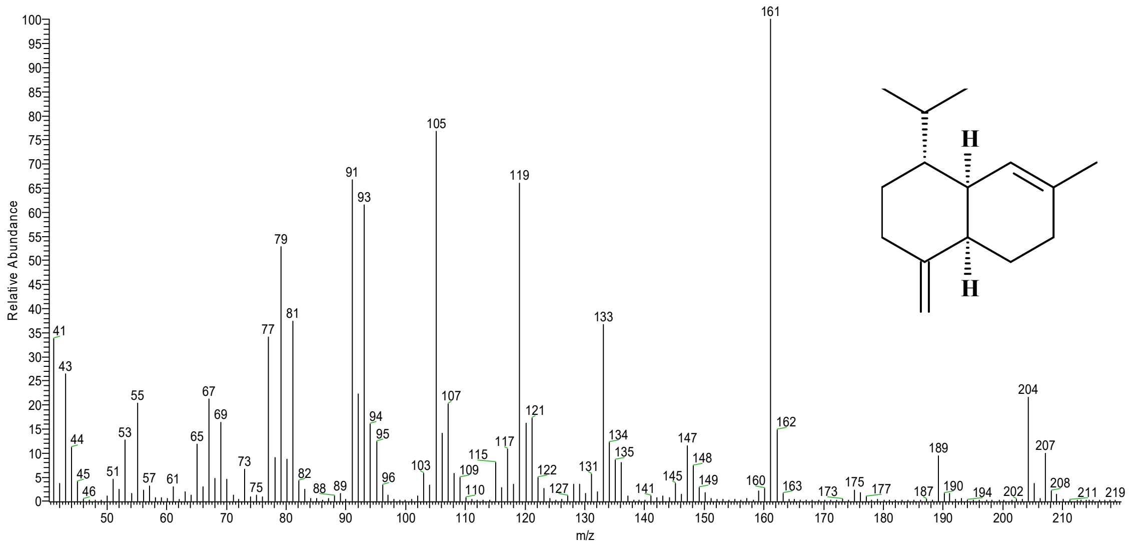

Figure S25. Mass spectrum of *γ*-muurolene (t_R 30.38 min).

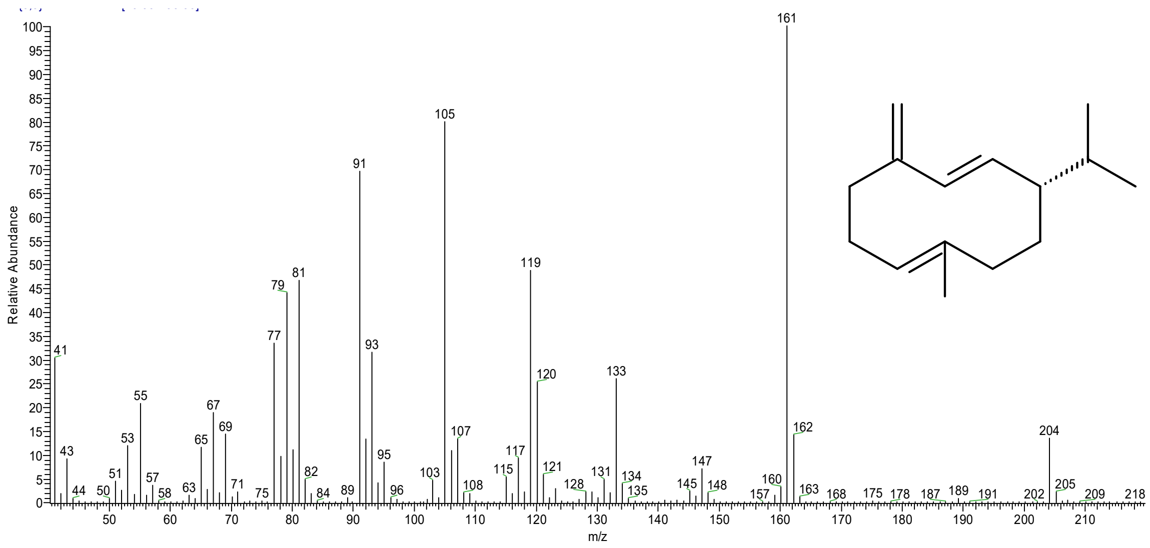

Figure S26. Mass spectrum of germacrene D (t_R 30.57; 30.58; 30.60 min).

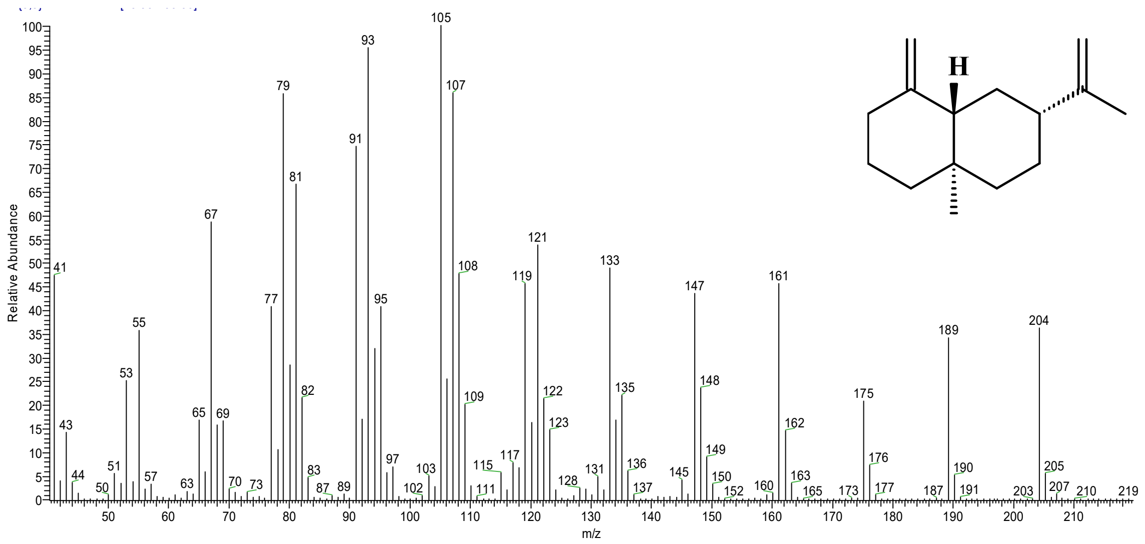

Figure S27. Mass spectrum of β-selinene (t_R 30.81; 30.82; 30.83 min).

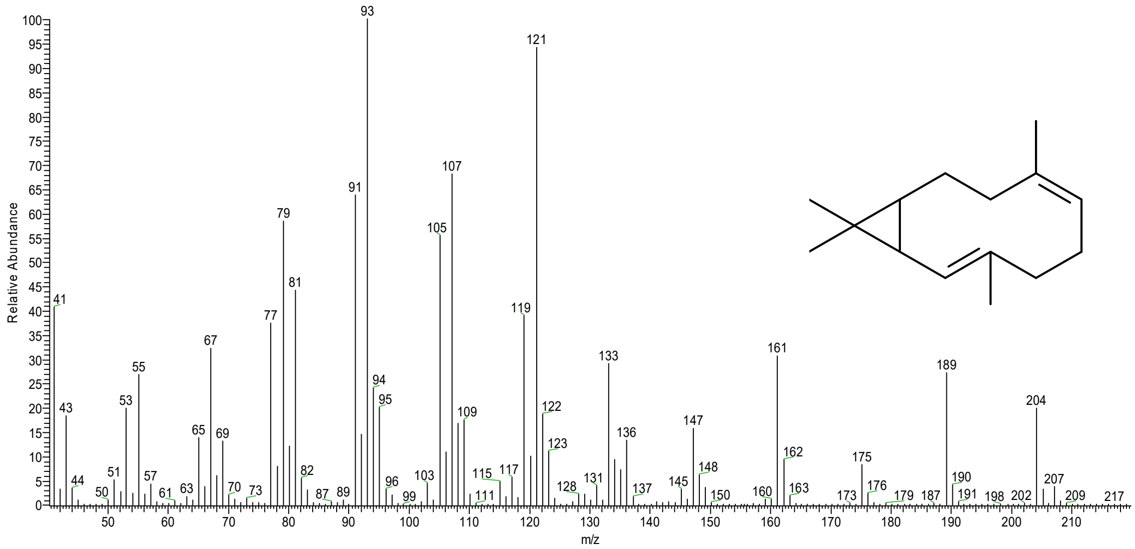

Figure S28. Mass spectrum of bicyclogermacrene (t_R 31.02; 31.03; 31.04 min).

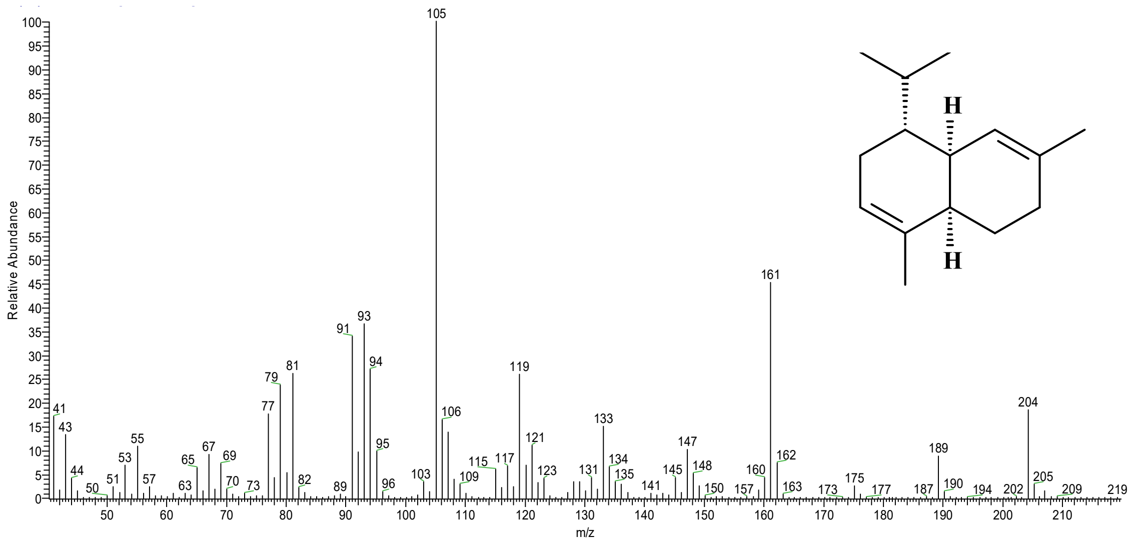

Figure S29. Mass spectrum of α-muurolene (t_R 31.10; 31.11; 31.13 min).

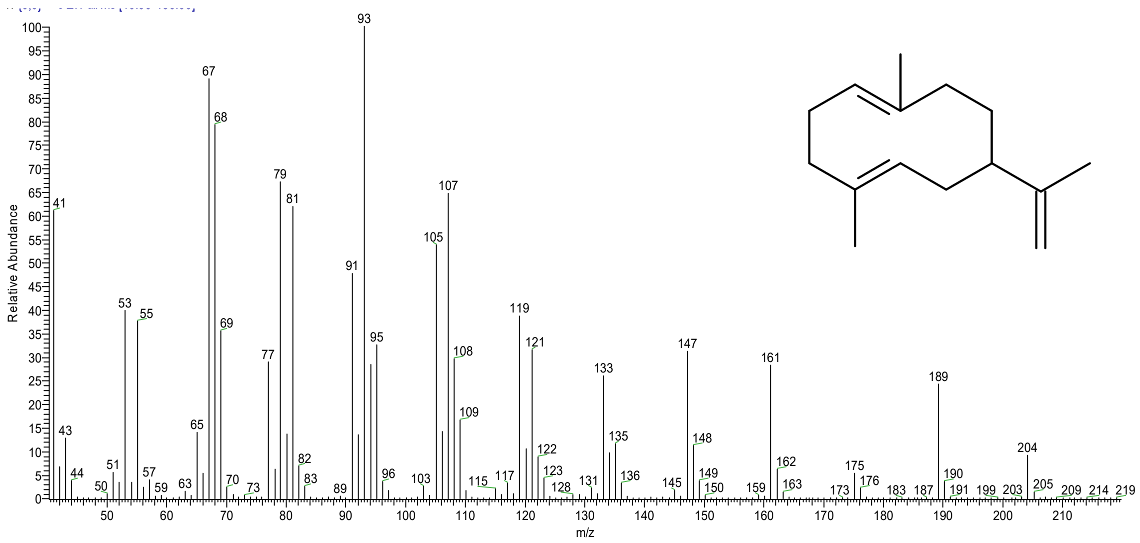

Figure S30. Mass spectrum of germacrene A (t_R 31.37; 31.39; 31.40 min).

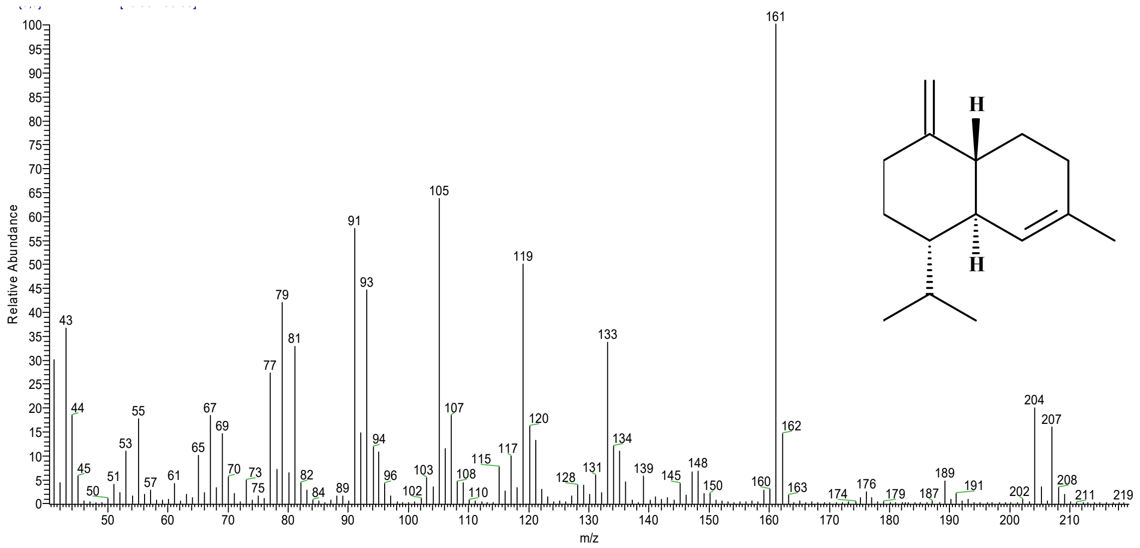

Figure S31. Mass spectrum of *γ*-cadinene (t_R 31.54; 31.55; 31.56 min).

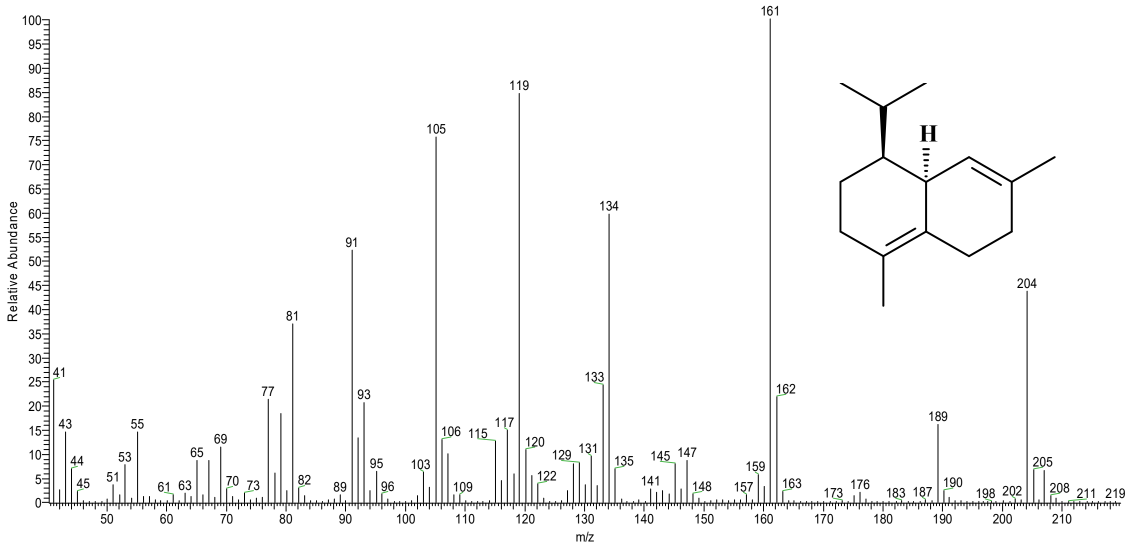

Figure S32. Mass spectrum of δ-amorphene (t_R 31.69; 31.70; 31.72 min).

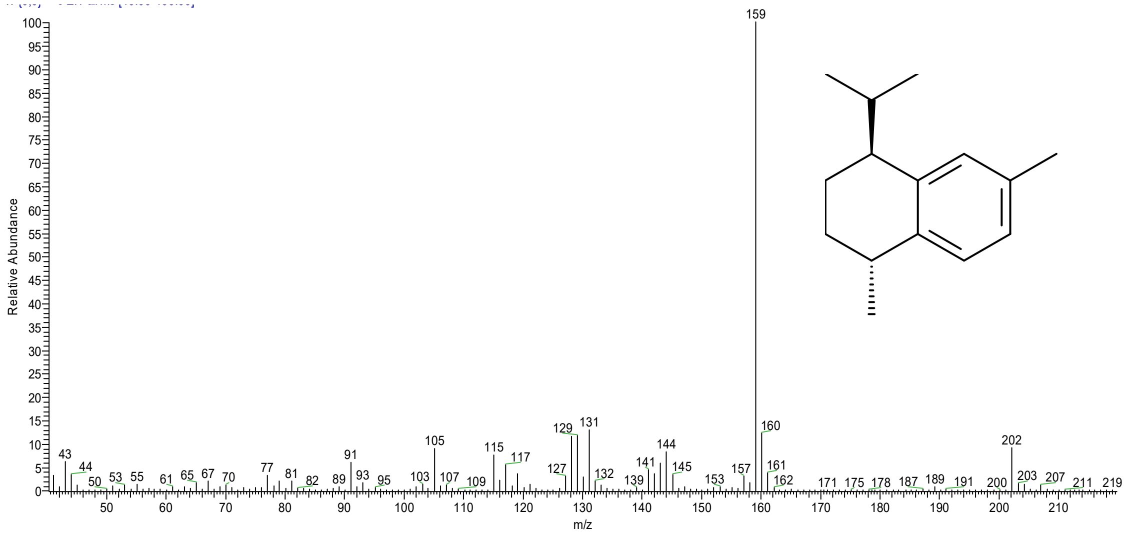

Figure S33. Mass spectrum of *trans*-calamenene (t_R 31.79; 31.82 min).

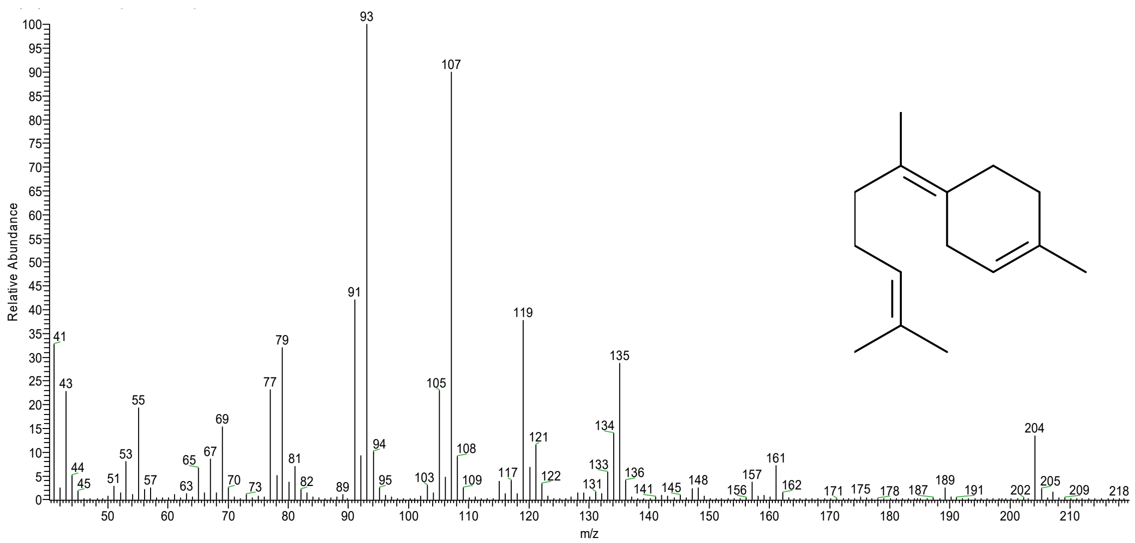

Figure S34. Mass spectrum of (*E*)-γ-bisabolene (tR 31.95; 31.96; 31.98 min).

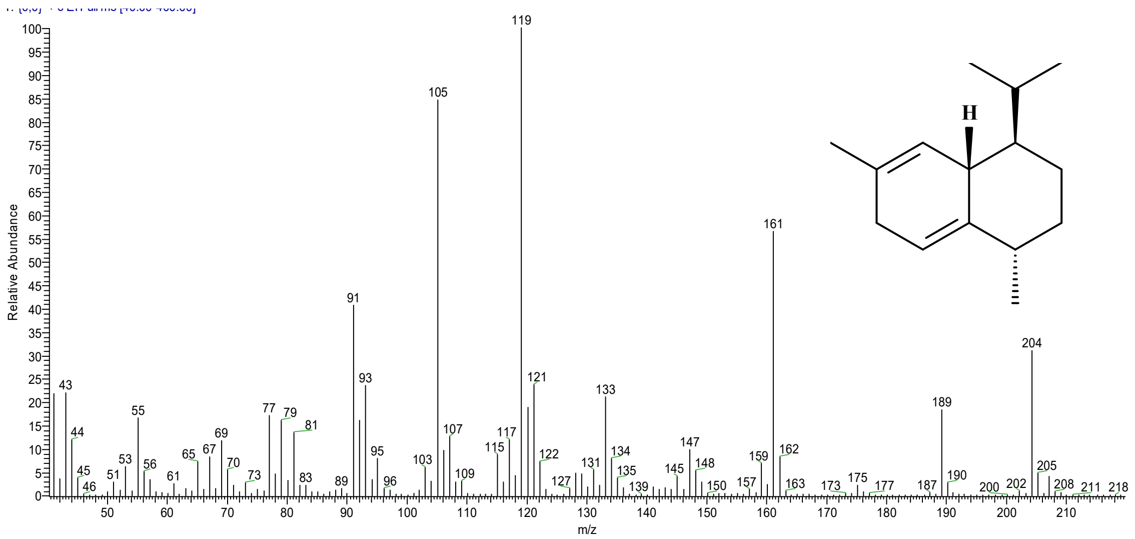

Figure S35. Mass spectrum of *trans*-cadina-1,4-diene (tR 32.12; 32.13; 32.14 min).

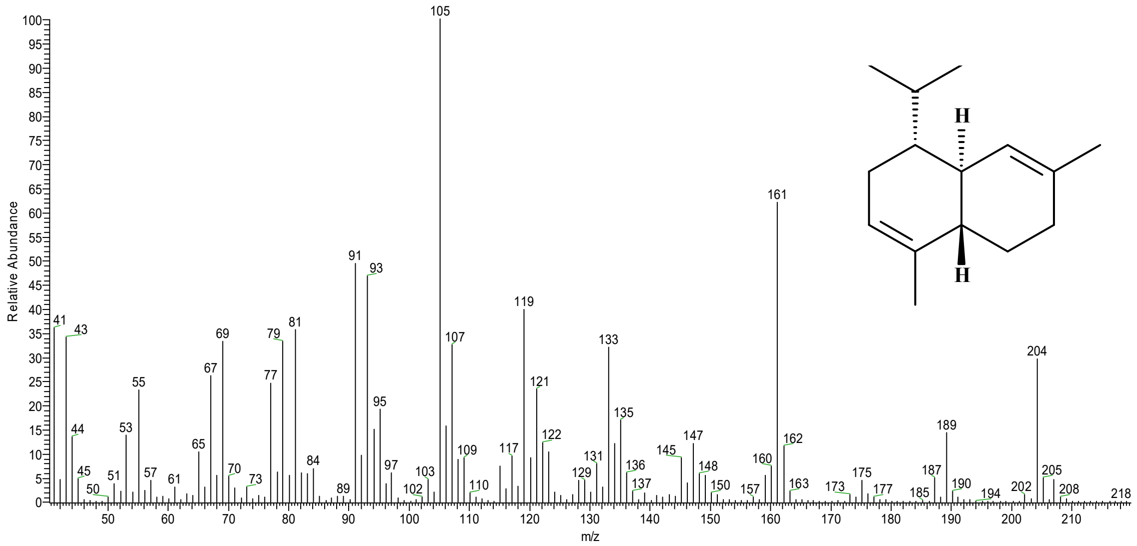

Figure S36. Mass spectrum of α-cadinene (t_R 32.25; 32.26; 32.28 min).

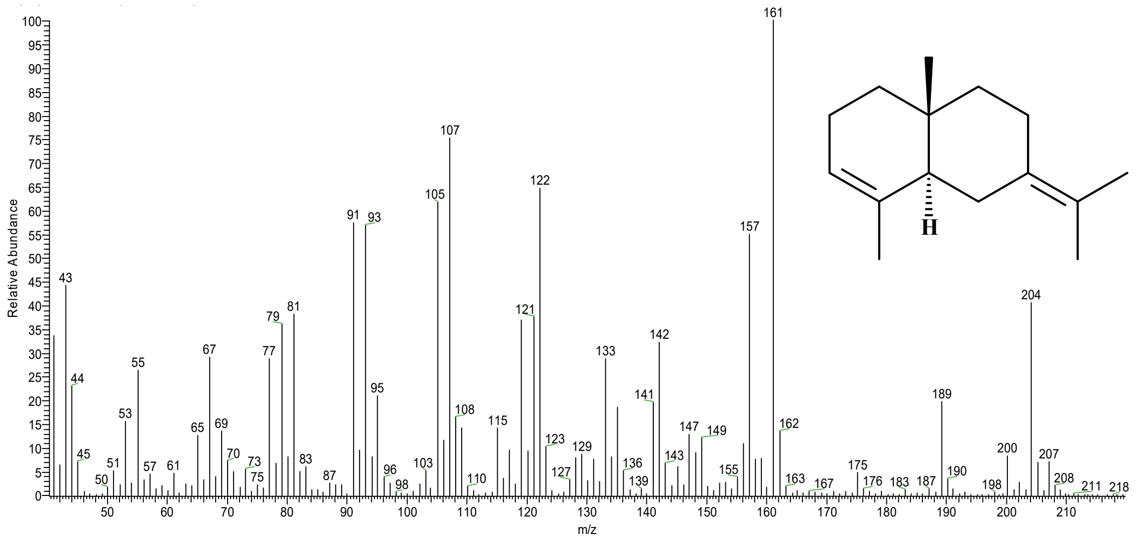

Figure S37. Mass spectrum of Selina-3,7(11)-diene (t_R 32.39; 32.40; 32.41 min).

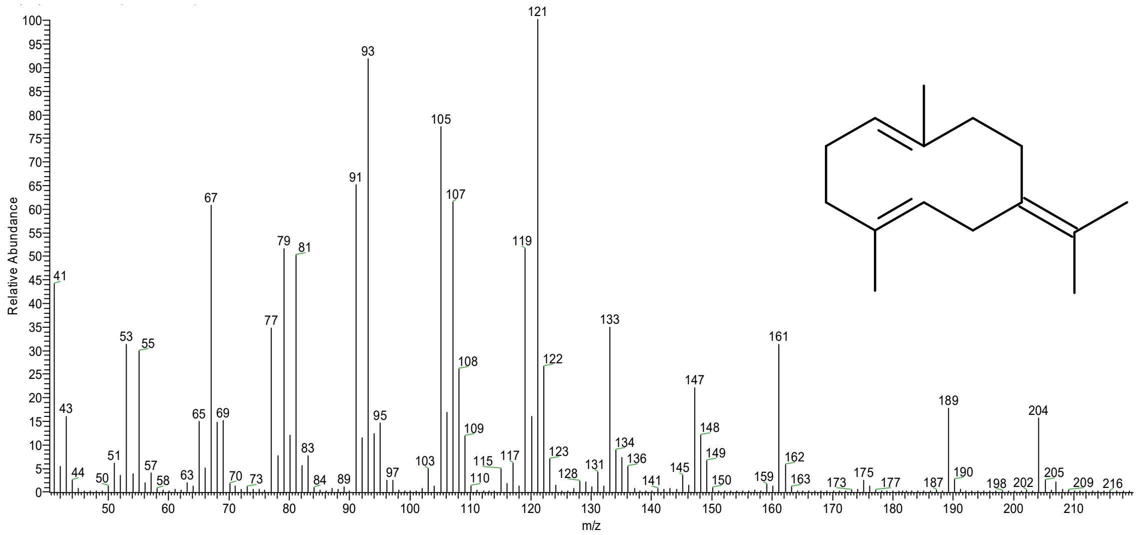

Figure S38. Mass spectrum of germacrene B (t_R 32.91; 32.92; 32.93 min).

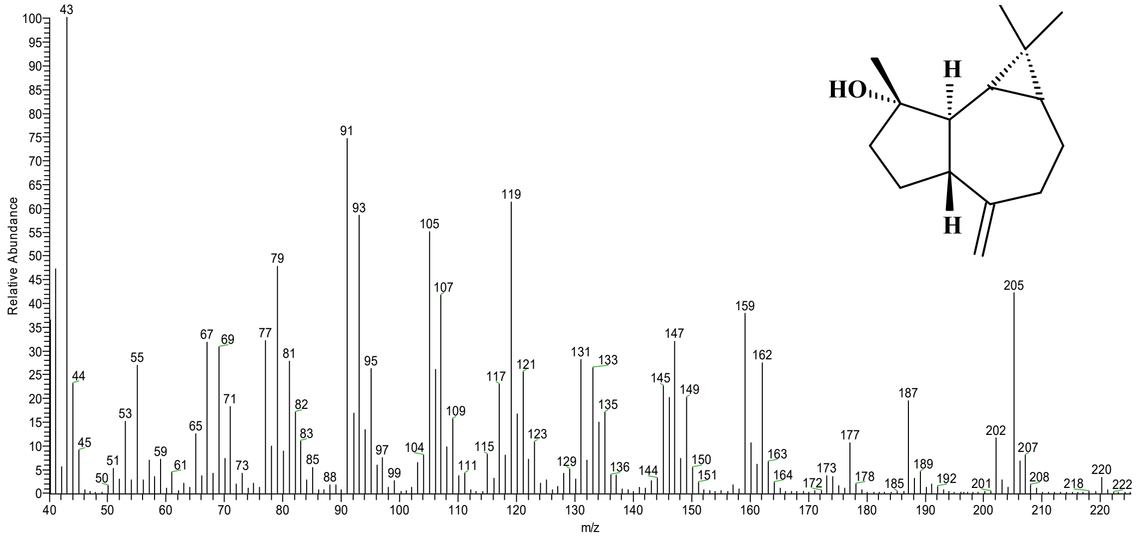

Figure S39. Mass spectrum of spathulenol (t_R 33.43; 33.45 min).

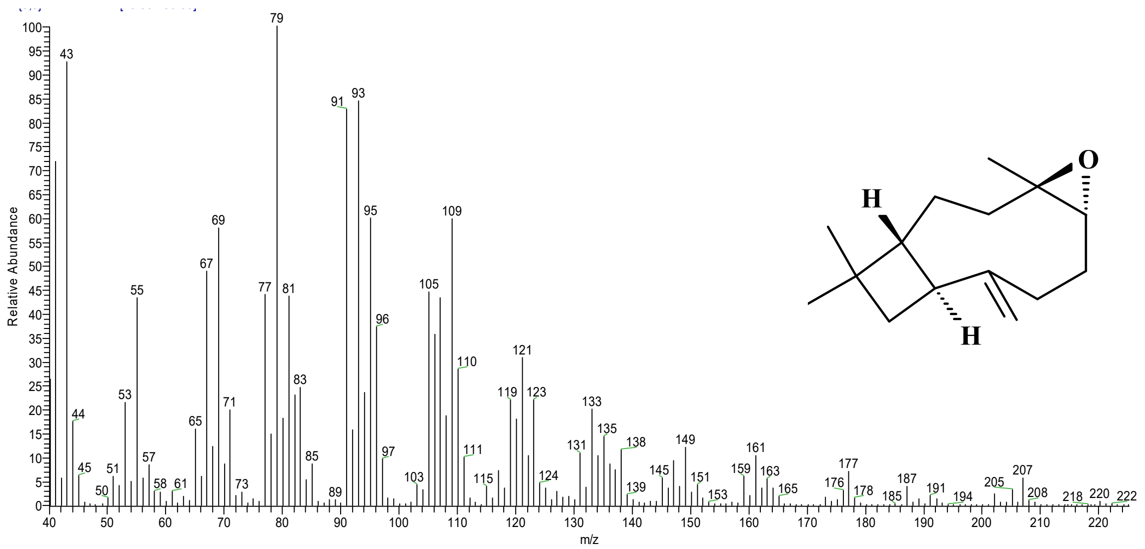

Figure S40. Mass spectrum of caryophyllene oxide (t_R 33.58; 33.59; 33.61 min).

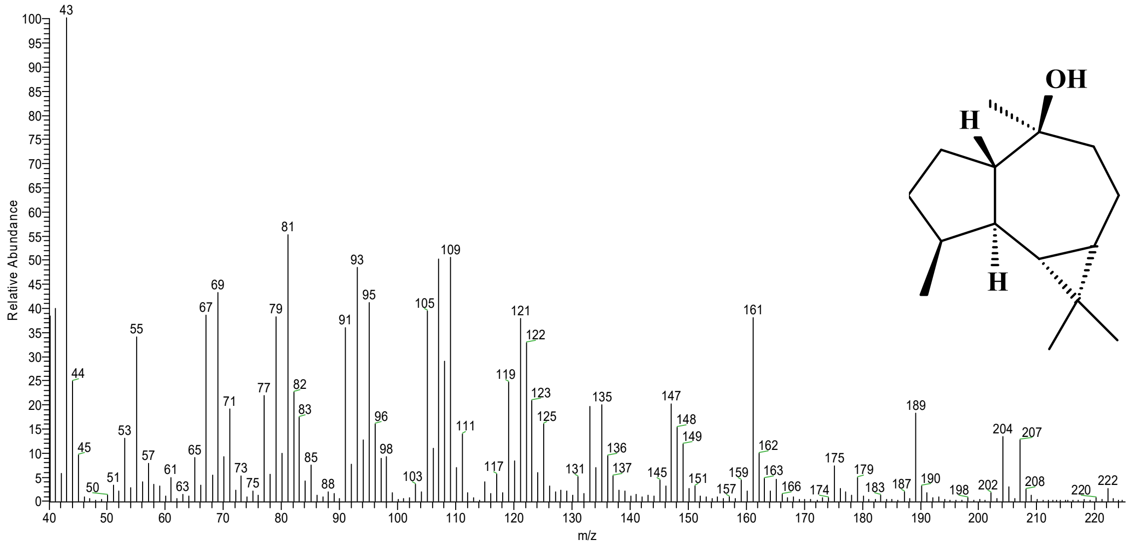

Figure S41. Mass spectrum of globulol (t_R 33.69; 33.70 min).

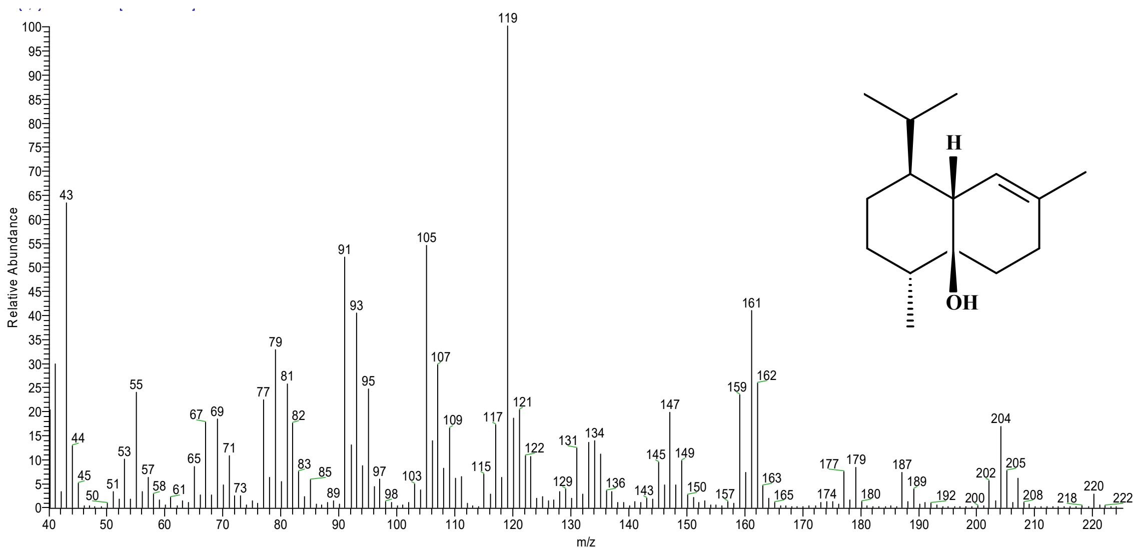

Figure S42. Mass spectrum of 1-*epi*-cubenol (t_R 34.91; 34.92 min).

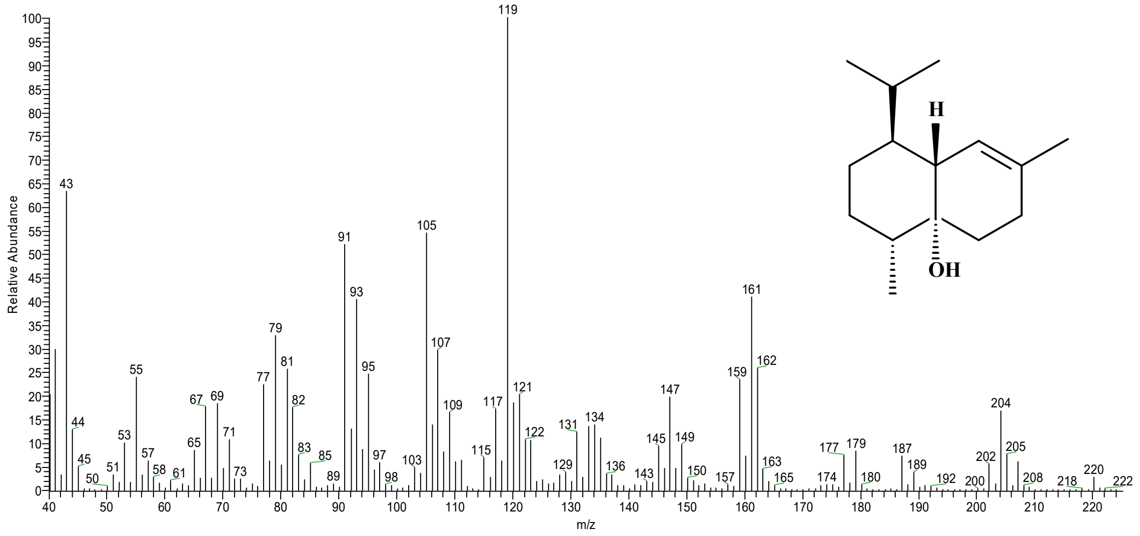

Figure S43. Mass spectrum of cubenol (t_R 35.35; 35.36 min).

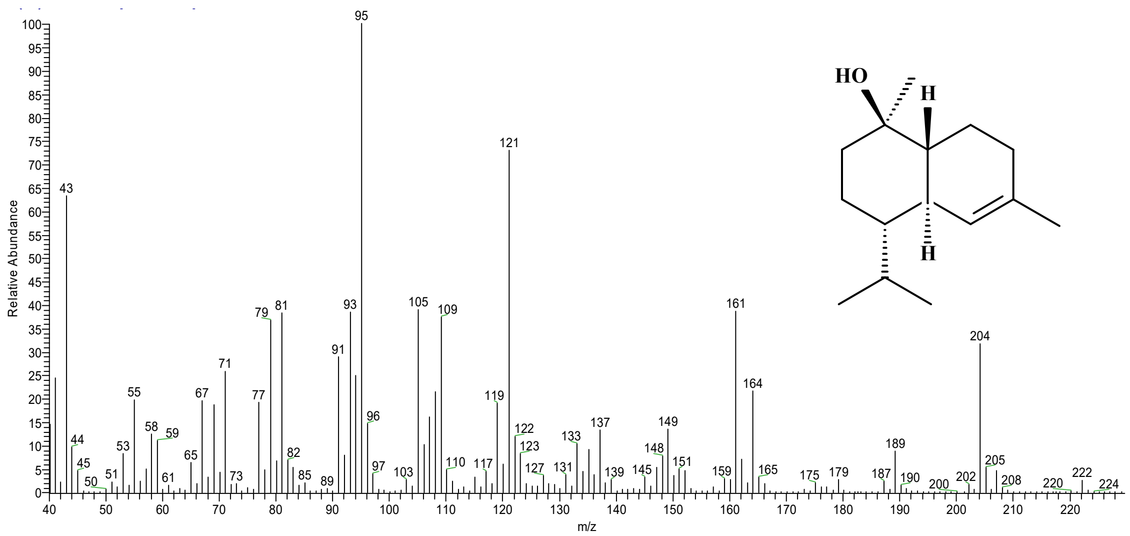

Figure S44. Mass spectrum of α-cadinol (t_R 35.67; 35.68; 35.70 min).

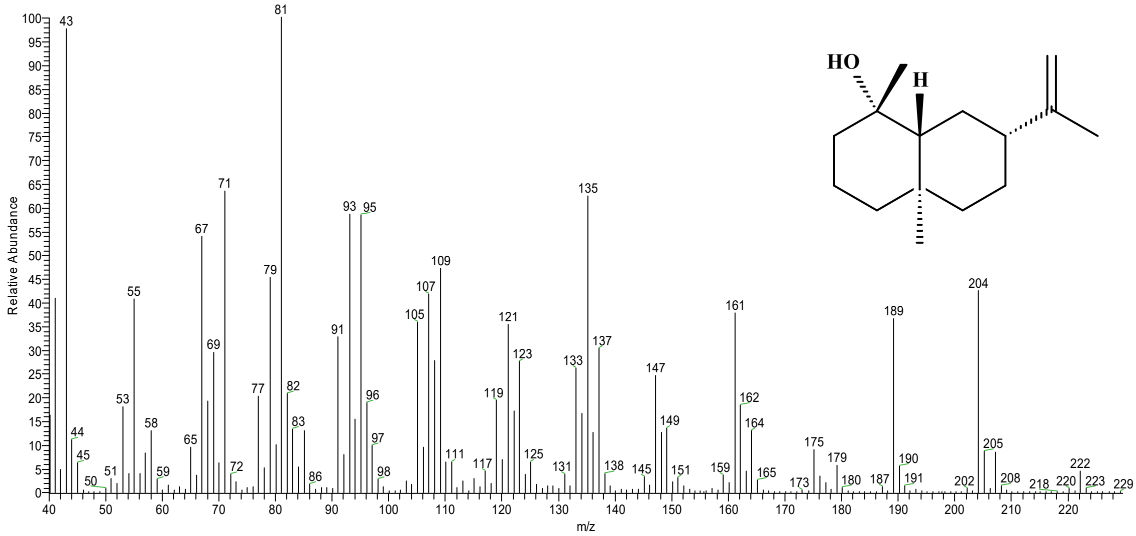

Figure S45. Mass spectrum of neo-intermedeol (t_R 35.76; 35.77; 35.79 min).

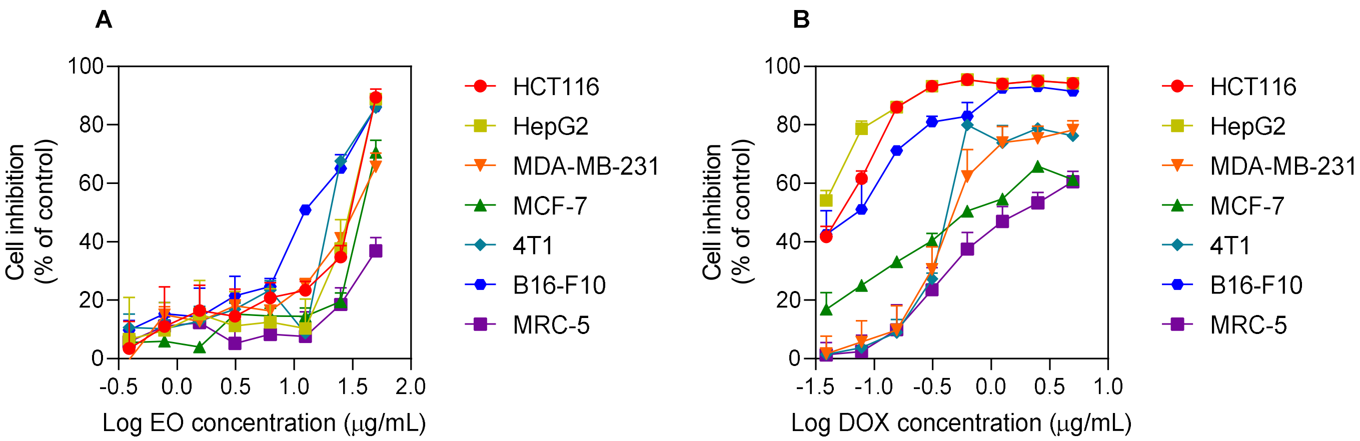

Figure S46. Concentration–response curves for cancerous and noncancerous cell lines treated with *A. neoinsignis* leaf EO (**A**) and doxorubicin (DOX, **B**). These data were obtained from at least three independent experiments performed in duplicate and measured via the Alamar blue assay after 72 h of incubation. Cancer cells: HepG2 (human liver cancer); HCT116 (human colon cancer); MCF-7 (human breast cancer); MDA-MB-231 (human breast cancer); 4T1 (mouse breast cancer); and B16-F10 (mouse melanoma) cells. Noncancerous cells: MRC-5 (human lung fibroblast) cells.

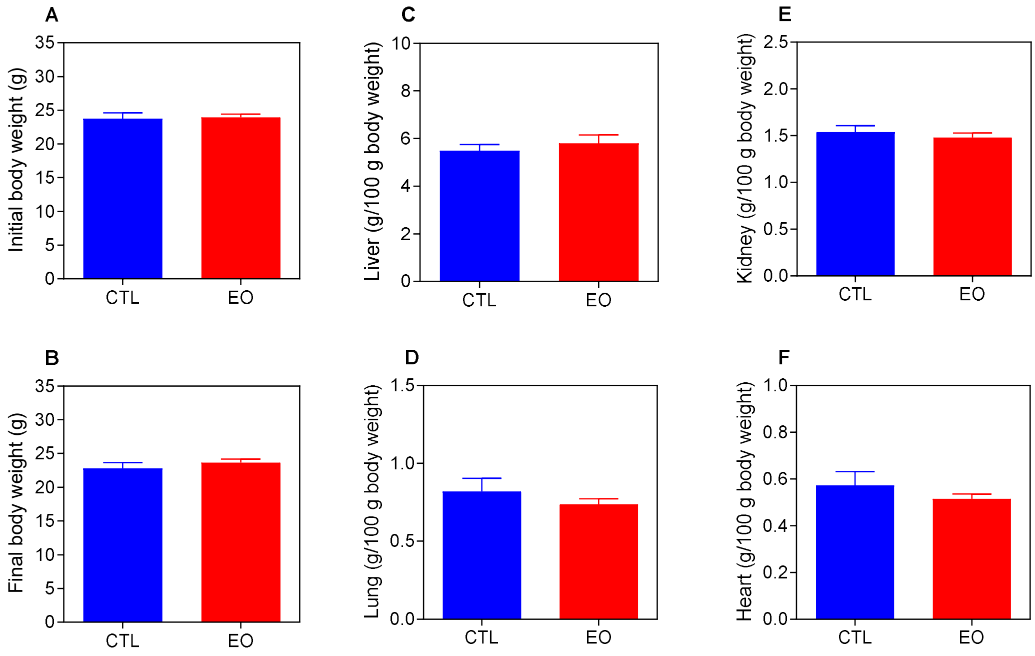

Figure S47. Effects of treatment with *A. neoinsignis* leaf EO on the body weight (**A** and **B**) and relative organ weight (**C, D, E** and **F**) of C.B.17 SCID mice bearing HepG2 cell xenografts. The treatments (40 mg/kg EO) were injected intraperitoneally into the mice daily for two weeks. Vehicle (5% DMSO) was used as a negative control (CTL). The data are shown as the means ± S.E.M.s of 8 animals.

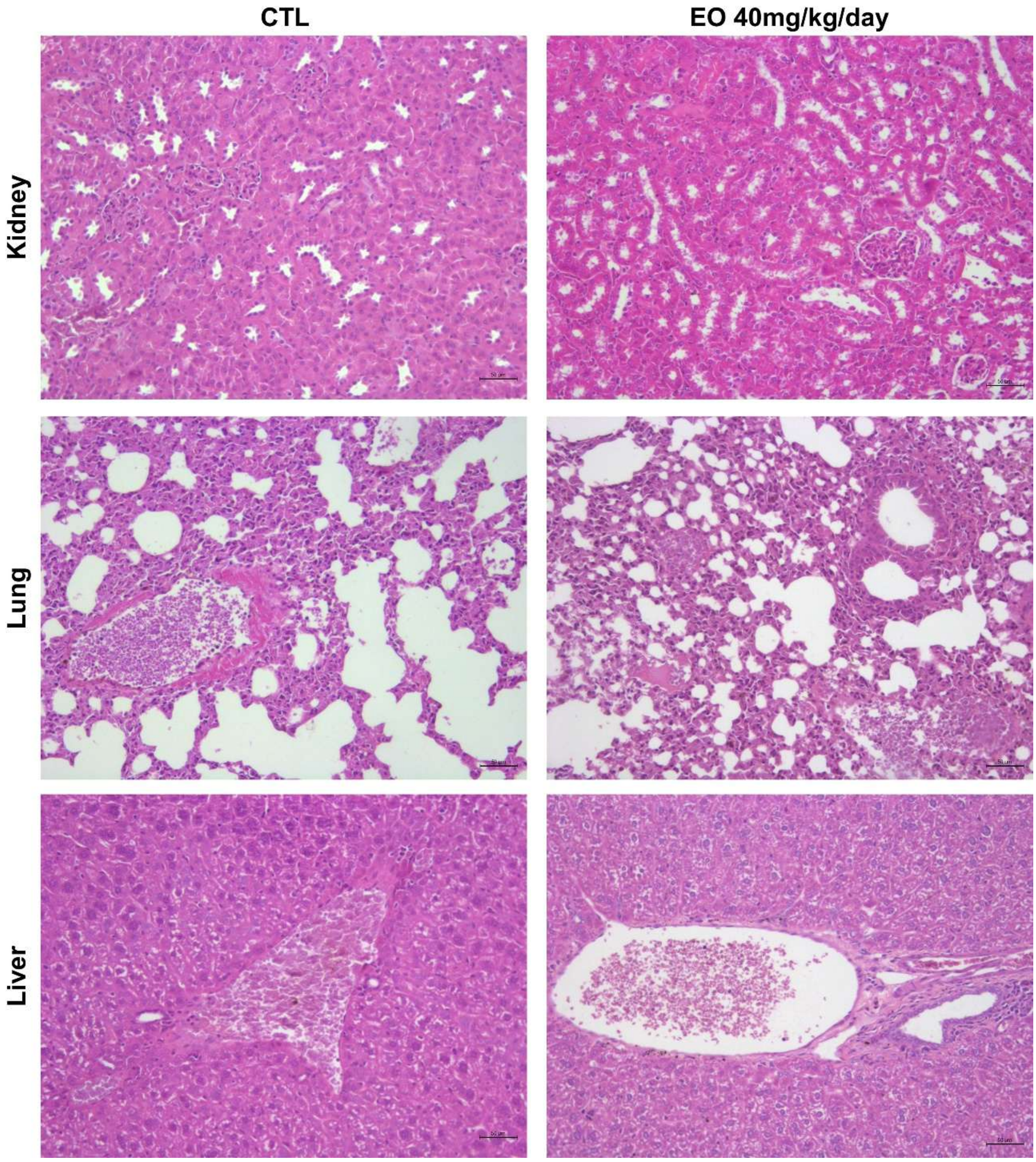

Figure S48. Representative photomicrographs of organs. The treatments (40 mg/kg EO) were injected intraperitoneally into the mice daily for two weeks. The vehicle (5% DMSO) was used as a negative control (CTL).
